# The Futures of Digital Democracy: Four Scenarios

**DOI:** 10.12688/openreseurope.22910.2

**Published:** 2026-06-04

**Authors:** Christian Fuchs, Joel Museba, Kevin Friesch

**Affiliations:** 1 Department of Media Studies, Paderborn University, Paderborn, North Rhine-Westphalia, Germany

**Keywords:** digital democracy, participatory digital democracy, representative digital democracy, participatory digital authoritarianism, representative digital authoritarianism, digital surveillance, dystopia, utopia, misinformation, fake news

## Abstract

This paper investigates the potential impacts of digitalisation on society and democratic governance through the construction of four realist science fiction scenarios. The study aims to delineate desirable and undesirable digital futures to inform policy discourse and strategic planning. It begins with the outlining of the research basis and methodology, where it employs a methodological synthesis of the Scenario Development Technique from the UN Strategic Foresight Guide and STEEP Analysis. This approach integrates a macro environmental assessment of socio-cultural, technological, economic, environmental, and political dimensions to identify key drivers of change and differences. These are then mapped onto a two-by-two matrix defined by two primary axes. This combination enabled the development of four scenarios examining the impact of digital technologies across these dimensions. The scenarios diverge along two axes. The vertical axis represents the mode of societal organisation and political power (authoritarianism versus democracy). The horizontal axis represents the mode of governance and citizen engagement (representation versus participation). By contrasting these divergent paths, this paper provides a critical framework for analysing how digital technologies intersect with political will and socioeconomic structures.

## Plain Language Summary

This paper explores how digital technology might change society and government in the future. To do this, the authors created four realistic science fiction scenarios based on and utopian and dystopian view. These stories are designed to help us think about what kind of digital future may be desired, and which ones should be avoided. The authors combined two research methods to create these scenarios:

•   UN Strategic Foresight: A tool for identifying big changes that will happen in the future.

•   STEEP Analysis: A way of looking at the big picture by studying five key areas: Society, Technology, Economics, Environment, and Politics.

•   Using this combination of methods, four scenarios are created and analysed:

•   Representative Digital Authoritarianism

•   Participatory Digital Authoritarianism

•   Representative Digital Democracy

•   Participatory Digital Democracy

By looking at these scenarios the paper then outlines possible future outcomes for the citizens and highlights the importance and weight of how technology can or cannot be used to augment politics.

## Introduction

The primary objective of this paper is to explore the unpredictable future of digital democracy and society by the year of 2050 through the lens of a realist science fiction approach. Four distinct speculative future scenarios are created and serve as imaginative tools for critical reflection.

This paper presents four scenarios of digitalisation and society with a special focus on the impacts of these developments on (digital) democracy. Methodologically, we combine the scenario technique and STEEP analysis in order to conceptualise four scenarios for the future of democracy in a digitalised society, as well as five societal dimensions of each scenario.

Given that society is a non-deterministic, complex system, we cannot predict how societies will look in 5, 10, 20, 50, or more years: “when systems are far from equilibrium, when they are bifurcating, small fluctuations can have great effects. This is one of the main reasons that the outcome is so unpredictable. […] in times of crisis and transition, the freewill factor becomes central. The world of 2050 will be what we make it. This leaves full rein for our agency, for our commitment, and for our moral judgment” ( Wallerstein, 1998, 64). Wallerstein writes that “which choice the participants collectively will make” about society’s future “is inherently unpredictable” ( Wallerstein, 2004, 77). The relative unpredictability is a phenomenon not limited to society. It is a feature of all complex dynamic systems, which is why the philosopher Wolfgang Hofkirchner (2011) says that the world is neither a determine clockwork nor an unpredictable cloud. He stresses that nature has “the freedom to choose between several alternatives which make up a non-devoid space of possibilities, compared with mechanical systems where there is only one possibility” ( Hofkirchner, 2011, 190). In 2000, we did not know that the COVID-19 pandemic would break out in 2020, Russia would invade Ukraine in 2022, or that OpenAI would launch ChatGPT in 2022 and become one of the most popular apps and Internet platforms.

Given the complexity and openness of the world, we do not know what digital societies will look like in 2050. We do not know if there will be a society by then, what societies will be democratic or undemocratic, and what roles digital technologies will play. Writing about digital society in 2050 is therefore not easy. Therefore, we have chosen fiction, or, to be more precise, realist science fiction, as a method for writing about the future of digital society and digital democracy. We present four science fiction scenarios of how digital societies could look in 2050. The method we employ is storytelling, namely the construction of four realist science fiction scenarios that aim at providing food for thought about what a future digital society could look like and what digital futures are desirable and undesirable. Fiction is “imaginative literature” ( Williams, 1983, 134). The beauty of fiction is that it presents stories that allow us to think about and imagine potential worlds and potential futures, and to thereby anticipate potential desirable and undesirable futures and think about what kind of (digital) society we want to live in and do not want to live in. Science fiction allows us to think about the immediate presence by engaging with potential (digital) futures.

What is science fiction? The science fiction writer Isaac Asimov gave the following definition: “Science fiction is that branch of literature which deals with a fictitious society, differing from our own chiefly in the nature or extent of its technological development” ( Asimov, 1953, 167). “SF texts imagine futures or parallel worlds premised on the perpetual change associated with modernity” ( Luckhurst, 2005, 3), especially changes having to do with science and technology. Science fiction is a form of fiction that draws on the existing knowledge and imaginative extrapolation of science and technology to explore the consequences for human beings and their societies. Given that utopias and dystopias play an important role in science fiction, the theme of digital democracy suits for science fiction storytelling well. This paper explores digital democracy and digital authoritarianism with the help of science fiction storytelling.

## 2. Democracy and authoritarianism

Fung and Wright (2003) distinguish between two forms of governance: top-down governance and participatory democracy. They introduce this differentiation as part of a classification of governance models:

• “In top-down governance structures decisions are made by actors at the peak of an organizational structure and then imposed on lower levels; in participatory governance, decisions involve substantial direct involvement of actors from the bottom tiers. [..] A shift of governance from the top-down adversarial to the participatory collaborative form involves the delegation of power from higher to lower levels of governance and to a broader array of participants” ( Fung & Wright, 2003, 261, 265).

We base one dimension, namely the x-axis, of our scenario analysis on Fung and Wright and distinguish between representative and participatory forms of governance. In representative forms of governance, a group of decision-makers decide on behalf of the population what should be done. In participatory forms of governance, there are mechanisms that engage affected individuals and groups living in society into decision-making processes that govern their lives.

The second dimension of our scenarios, the y-axis, concerns the basic mode of society’s organisation. We here draw a distinction between democracy and authoritarianism, democratic and authoritarian societies.

One starting point for concepts of autocracy is Aristotle’s typology of politics (Aristotle, 2013, books 4 & 5). He distinguishes between good and bad forms of politics, where one person (monarchy, tyranny, etc.), a few people (aristocracy, oligarchy, etc.), and many rule (polity, democracy, etc.). The basic distinction of political systems Aristotle established is the one between the rule by one person, the few, and the many.

Our model’s two dimensions, the distinction between democracy/authoritarianism and between participation/representation, reflect fundamental questions of political theory, namely governance and society’s mode of organisation. The next section is concerned with the distinction between democracy and authoritarianism. The section “Four scenarios of digital futures” addresses the distinction between representation and participation in democratic theory.

### Democracy

Democracy has different dimensions. It is an idea and a set of principles (“democratic”), a group of people (“democrats”, citizens, etc.), a type of action (“democratising”, “democratic practices”, “struggles for democracy”, etc.), and an institution and system (“democracy”). Taken together, this means that democracy is not static but a dynamic process in which humans, based on a set of constitutional democratic principles, engage in practices that result in decisions that are binding for all and represent the collective will of those participating.

Understandings of democracy have in common that they conceive of democracy as the self-government of human beings (see, for example, Crick, 2002; Dahl, 2006, and Held, 2006). Democracy stands opposed to monarchies (rule by a single monarch), oligopolies and aristocracies (rule of the few), as well as dictatorships and tyrannies (rule through violence and terror). Democracy is not just a means for minimising domination but also an attempt to minimise the rule by violence.

In modern societies, citizens who hold the citizenship of a particular nation-state have been conceived as the subjects of democracy. Nation-states continue to be important organisational forms of politics. However, societies have become more transnational and, besides local, regional, and national characteristics, also feature supranational organisations, transnational communication networks, and transnational flows of money, goods, power, humans, data, and knowledge. Societies are today both national and transnational. Therefore, we need to think of politics and democracy as operating at various organisational levels.

The concept of democracy itself is broadly contested. Consequently, when defining democracy, scholars differentiate between its various concrete manifestations and abstract conceptions, i.e. models of democracy. A model of democracy is a specification of the general features of democratic systems as well as the clarification of what features are central in a particular model (see Macpherson, 1977 and Held, 2006). There is a variety of models of democracy. In our own approach, we identify six models of democracy: (a) constitutional democracy, (b) representative democracy, (c) direct democracy, (d) deliberative democracy, (e) participatory democracy, and (f ) pluralist democracy ( Fuchs, 2025). Currently existing democracies often combine different models and dimensions of democracy.

In the model of constitutional democracy, there is a stress on the importance of constitutions that protect civic and human rights and democracy’s central feature. Representative democracy emphasises the election of political representatives who govern society through free, fair, regular, universal, equal, secret, and transparent elections as democracy’s central feature. In direct democracy, referenda and citizens’ initiatives, where citizens directly decide and vote on key societal topics, act as central features. In deliberative democracy, open, inclusive, egalitarian, non-manipulative, non-coercive political debates in the public sphere present democracy’s key feature. Deliberative democracy stresses the importance of political communication within democratic institutions, between citizens, and between governments and citizens, which includes aspects such as citizen-to-citizen debates, a pluralist media and news reporting landscape (news media diversity), social movements and a vivid civil society, and parliamentary debates. In participatory democracy, the self-management of organisations, democracy beyond elections, and the mobilisation of resources that enable democracy are democracy’s key aspects. Pluralist democracy emphasises the plurality of parties, political groups, political ideas, political voices, political visibility, political interests, and taking minority interests seriously as democracy’s key features.

We define digital democracy as the use of digital media in the practice of democracy. It refers to the digital mediation of democracy. Digital democracy has three encapsulated symbolic dimensions: the production and sharing of democratic information (digital democratic information), democratic communication (digital democratic communication), and the co-creation of democracy (digital democratic co-production) ( Fuchs, 2025). Models of democracy, together with the affordances and features of digital technology, enable dimensions of digital democracy. They include constitutionalist digital democracy, representative digital democracy, direct digital democracy, pluralist digital democracy, deliberative digital democracy, and participatory digital democracy (see Fuchs, 2025).

### Authoritarianism

In academic discourse, the notion of authoritarianism was developed in the 1920s, 1930s, and 1940s by authors such as Wilhelm Reich, Erich Fromm, Theodor W. Adorno, and Franz L. Neumann ( Adorno *et al.*, 1950; Fromm, 1936; Fromm, 1942/2001; Fromm, 1984; Neumann, 1944/2009; Reich, 1933/1972). They wanted to understand how Nazism worked and how it managed to appeal to a large number of citizens. Their starting point was the individual’s personality, which is why they spoke of the “authoritarian personality” ( Adorno *et al.*, 1950). The influence of authoritarian fathers, families, bosses, and politicians on the individuals‘psyche and on society shapes the authoritarian. But authoritarianism has, for these authors, not been a purely individual phenomenon. Rather, they argued that it extends from the individual to society, which is why they spoke of the “authoritarian society” ( Fromm, 1955/1991, 148; Reich, 1933/1972, 21), “the authoritarian system” ( Fromm, 1942/2001, 18), the “authoritarian structure of society” ( Neumann, 1944/2009, 150), or the “authoritarian order” ( Neumann, 1944/2009, 128).

Authoritarianism involves not only dictatoral rule but also violence, namely, “the passion to destroy life and the attraction to all that is dead, decaying, and purely mechanical” ( Fromm, 1973/1997, 6). Authoritarian societies love the “act of dismemberment” (329), “to destroy for the sake of destruction” (186), and “to tear apart living structures” (329). Authoritarian societies are what Fromm (1973/1997) calls ‘necrophilic societies’. They are not resilient and life-affirming but have a love for death and destruction. Several researchers have conducted empirical research on how to measure right-wing authoritarianism (see, for example, Adorno *et al.*, 1950; Altemeyer, 1981, and Meloen, 1993).

For these classical authors, studying authoritarianism required an interdisciplinary approach that brings together the fields of political economy, sociology, critique of ideology, and psychology in order to understand the various levels of authoritarianism and their interaction. Therefore, classical studies of authoritarianism provide the important insight that understanding this phenomenon requires an interdisciplinary approach.

Also in contemporary academic discourse, there are approaches that understand authoritarianism as a type of society (for example, Fuchs, 2018a; Giroux, 2015, and Wiatr, 2019). Fuchs stresses that authoritarianism includes psychological, political, economic, and cultural-ideological elements and has a variety of organisational levels: “[It] can take place at the level of the individual, group, institution or society as a whole” ( Fuchs, 2018a, 58).

In the debate on authoritarianism, there is the widespread assumption that we have been witnessing the return of authoritarianism in society in the context of multiple crises. Therefore, terms such as the new authoritarianism ( Giroux, 2015 and Wiatr, 2019), third wave of autocratisation ( Lührmann & Lindberg, 2019), post-democracy ( Crouch, 2004), late fascism ( Toscano, 2023), neoliberal fascism ( Giroux, 2019), new fascism ( Traverso, 2019), or post-fascism ( el-Ojeili, 2018; Tamás, 2000) have been created.

Authoritarianism maintains an ambivalent relationship to the media. It tends to question and repress critical and independent media, oppose media plurality, and use media as a tool of propaganda. Given that media today is largely digital and media is an aspect of the public sphere, as part of discussions of new authoritarianism, concepts and analyses of digital authoritarianism have also emerged.

New authoritarian forces have used the internet and social media as means of propaganda, disinformation, and information warfare (see European External Action Service, 2023; European External Action Service, 2025). In the light of foreign information manipulation and interference (FIMI), the European Commission has advanced plans for the European democracy shield and advancing digital sovereignty ( Special Committee on the European Democracy Shield, 2025).

In the light of the digitalisation of authoritarianism context, authors have spoken of digital authoritarianism ( Gosytonyi, 2023; Maerz, 2024; Pearson, 2024, and Roberts & Oosterom, 2024), digital autocracy ( Frantz *et al.*, 2020 and Sinpeng & Koh, 2022), digital populism ( Bartlett *et al.*, 2011 and Gerbaudo, 2024), digital fascism ( Degeling, 2024; Demír, 2025; Fielitz & Marcks, 2019, and Fuchs, 2022), and networked authoritarianism ( Mackinnon, 2011). It has also been debated whether it is better to speak of digital populism or digital authoritarianism ( Fuchs, 2018b and Gerbaudo, 2018). “I suggest that we redescribe DA [digital authoritarianism] as any situation where digital technologies systematically promote authoritarian politics” ( Pearson, 2024, 13). “Digital authoritarianism is broadly defined as a set of digital practices which are aimed at harming democracy” ( Maerz, 2024, 210). “Classical fascism used stormtroopers and monopolised, state-controlled broadcast media (such as the Volksempfänger). Contemporary fascism, among other means, uses troll armies and social media in order to attack defined enemies. Classical fascism was strictly organised top-down based on the leadership principle. Contemporary fascism fetishises the leader and more combines fascist leadership with networked, decentralised organisation” ( Fuchs, 2022, 321).

Such approaches share the understanding that digital authoritarianism consists of the use of digital media in the practice of democracy. It refers to the digital mediation of authoritarianism. Digital authoritarianism has three encapsulated symbolic dimensions: the production and sharing of authoritarian information (digital authoritarian information), authoritarian communication (digital authoritarian communication), and the co-creation of authoritarianism (digital authoritarian co-production).

Scenarios that analyse potential futures of democracy, society, and technology should address what role (digital) authoritarianism and/or democratic resilience against (digital) authoritarianism play.

## Writing about the future

Our approach to conceptualising scenarios for the future development of digital democracy and society adopts the typology presented by Raymond Williams (1978/2010). Raymond Williams (1921–1988) was a literary and cultural theorist, a pioneer of cultural studies, and a novelist. He made crucial contributions to contemporary cultural sociology. As a significant intellectual figure of the twentieth century, his work influenced thinkers as diverse as Stuart Hall, Edward Said, and Jürgen Habermas ( Milner, 2003, 199). Crucially for our purposes, as Andrew Milner (2016) points out, Williams maintained a sustained and serious engagement with utopia, dystopia, and science fiction throughout his career, publishing scholarly work specifically addressed to these genres and even authoring two future-oriented novels ( Milner 2016, 416). It is this combination of sociological, cultural and political theory with speculative and fictional modes of writing about the future that makes Williams’s typology a particularly productive framework for the digital democracy scenarios developed in this paper. Williams distinguishes between eight ways of writing about the future. Table 1 shows his typology. He identifies four different story types that either have an optimistic (utopian) or a pessimistic (dystopia) character, which results in a total of eight different types of how to tell stories about the future.

**Table 1.** Raymond Williams’ (1978/2010, 95) typology of ways of writing about the future.

	Utopia	Dystopia
Paradise and hell	Paradise: “a happier life is described as simply existing elsewhere”	Hell: “a more wretched kind of life is described as existing elsewhere”
Externally altered world	External Utopias: “a new kind of life has been made possible by an unlooked for natural event;”	External Dystopias: “a new but less happy kind of life has been brought about by an unlooked for or uncontrollable natural event”
Willed Transformation	Human-Centred Utopias “a new kind of life has been achieved by human effort”	Human-Centred Dystopias: “a new but less happy kind of life has been brought about by social degeneration, by the emergence or re-emergence of harmful kinds of social order, or by the unforeseen yet disastrous consequences of an effort at social improvement”
Technological Transformation	Technological Utopias “a new kind of life has been made possible by a technical discovery.”	Technological Dystopias “the conditions of life have been worsened by technical development.”

Williams identifies eight ways of writing about the future. He distinguishes between utopias and dystopias, stories about and descriptions of a better and a worse world. In addition, he distinguishes descriptions of future society according to the type of transformation that has taken place. First, some stories play in completely different worlds (for example, on another planet), and these are often highly unrealistic and fantastic in that they are either perfect worlds without problems or nightmares. He calls these scenarios “paradise” and “hell”. There is often no trace of contemporary power structures in such stories. Second, there are stories in which a natural disaster or radical event has completely transformed society. Examples are earthquakes, volcanic eruptions, or floods. Third, there are descriptions of a future society in which humans have wilfully transformed society. Fourth, there are futures where the application of science and technology has fundamentally altered life. Combining the four types of transformation and the two moral political economies (utopia, dystopia) as criteria of distinction yields in eight types of writing about the future.

Williams has a particular interest in human-centred and technological utopias and dystopias. For us, the combination of both holds special interest. The technological dimension is interesting for us because we intend to write about digital futures. Human-centredness is important because we are interested in writing about the future of human society on Earth. Therefore, there are no extraterrestrial beings such as Martians in our scenarios, and we focus on life on Earth and not on other planets. Human-centred stories also allow us to focus on potential futures of contemporary power structures. In our scenarios, power structures continue to exist but have been altered by humans wilfully as the outcome of certain social struggles. Therefore, our scenarios are manifestations of the lower part of Table 1 that focuses on human-centred utopias and dystopias as well as technological utopias and dystopias. Our digital futures scenarios ask what happens when we combine the technological and the human-centred storytelling with the question of how the futures of digital society could look.

For Erik Olin Wright, real utopias are neither fantasies of the impossible nor fetishisations of pragmatism. Rather, real utopias are “utopian ideals that are grounded in the real potentials of humanity, utopian destinations that have pragmatically accessible waystations, utopian designs of institutions that can inform our practical tasks of muddling through in a world of imperfect conditions for social change” ( Wright, 2003, viii). Our scenarios are real utopias and real dystopias. They are possible futures grounded in the real positive and negative potentials of contemporary digital society.

## Methodology

### The scenarios technique

We are using the scenarios technique as a method for thinking about the future of (digital) democracy. What are scenarios?

• “Scenarios are provocations to broaden our understanding of how the future may evolve, which allows us to prepare not for one but multiple ways the future might unfold. In the foresight process, scenarios are generated by identifying emerging drivers of change that may affect the future” ( UN Futures Lab, 2023. 1).

The critical uncertainties method is one method for developing scenarios: “Four distinct scenarios are framed by placing two prioritised highly-critical and highly-uncertain trends or drivers of change on a two-by-two matrix. The two axes represent the two extremes of the two drivers of change” ( UN Futures Lab, 2023, 1). We use this method for identifying four scenarios.

We used the critical uncertainties method for creating a Cartesian system with two axes that combine the mode of societal organisation as one axis and the form of governance as the other axis.

### Four scenarios of digital futures

**Figure 1.**
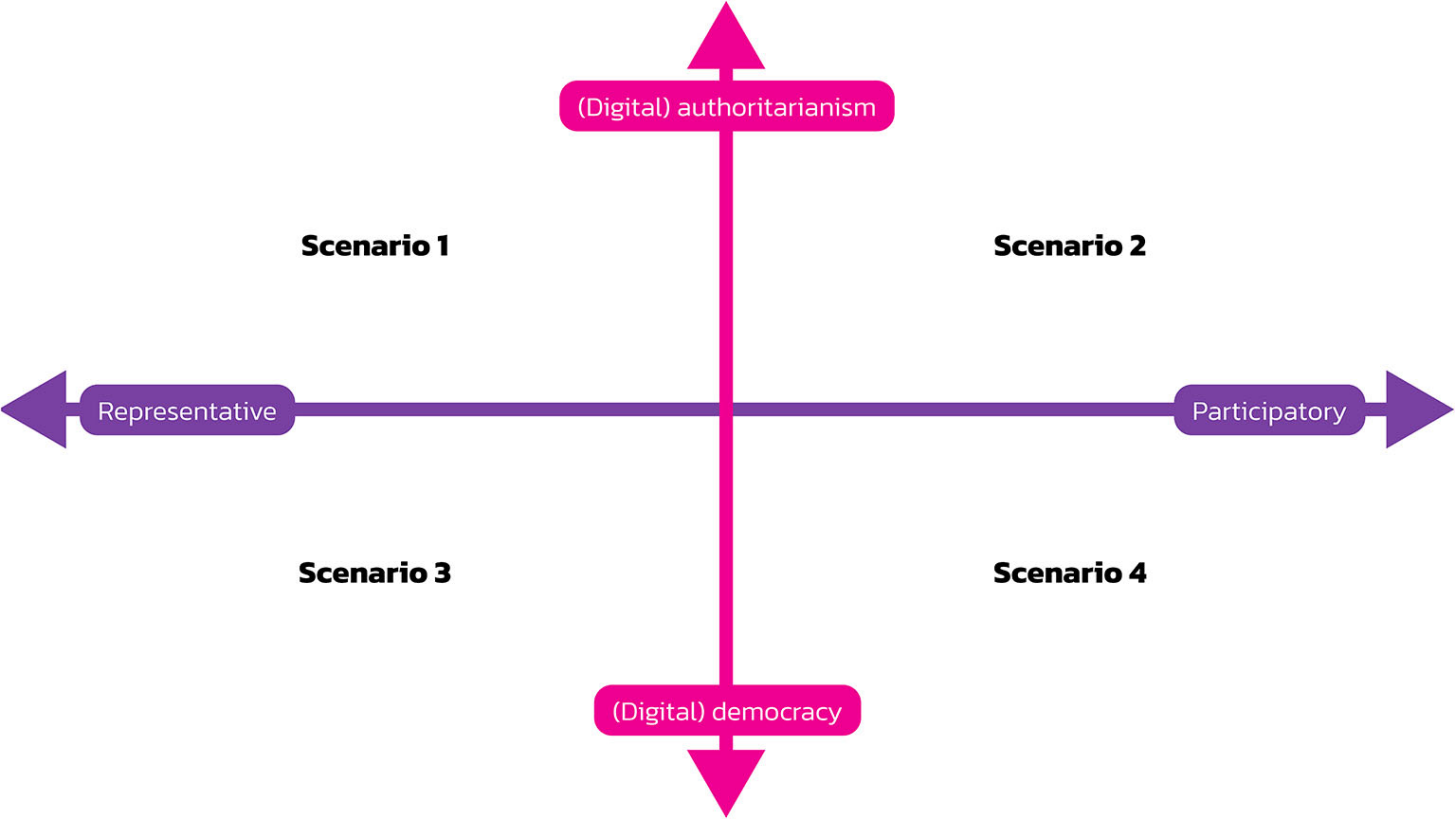
Four scenarios for digital society’s future.

Combining the two axes of governance (representation vs. participation) and societal organisation (authoritarianism vs. democracy) results in the model visualised in Figure 1. Its four quadrants represent four scenarios for the future of digital society.

The four scenarios have the following names:

• Scenario 1: Representative Digital Authoritarianism
• Scenario 2: Participatory Digital Authoritarianism
• Scenario 3: Representative Digital Democracy and Society
• Scenario 4: Participatory Digital Democracy and Society.

Representative digital authoritarianism is a dictatorship with a strong leader and a one-party system that claims to represent the people’s interests and uses digital technologies as a means of control. Participatory digital authoritarianism is a dictatorial system ruled by a strong leader. It features a democratic facade that uses digital technologies for organising plebiscites to retain citizens’ approval of the regime. Via digital media, the regime co-opts citizens and asks them to participate in a dictatorship. Representative digital democracy and society is a system where authorities use digital technologies for e-government, open governance, and e-voting. Participatory digital democracy and society is a system where citizens use digital technologies for various forms of citizen engagement in politics and society.

In other work, we identified six dimensions and versions of digital democracy ( Fuchs, 2025):

• Constitutional digital democracy:

Constitutional digital democracy encompasses legal, social, and technical measures and procedures aimed at safeguarding human rights and dignity in online spaces.

• Representative digital democracy:

Representative digital democracy involves using digital media to organise the election of representatives to democratic parliaments, assemblies, and councils, as well as to facilitate interaction between citizens on one side and governments and elected representatives on the other.

• Direct digital democracy:

Direct digital democracy involves using digital media to hold plebiscites, where citizens vote directly on particular topics, policies, and draft legislation.

• Pluralist digital democracy:

In a pluralist digital democracy, measures are implemented to prevent the tyranny of the majority in e-voting and to promote the inclusion of minorities in democracy, ensuring the presence of multiple voices and a diverse political landscape.

• Deliberative digital democracy:

Deliberative digital democracy entails utilising digital media to promote online debates within the public sphere, activities of civil society, and democratic journalism and news.

• Participatory digital democracy:

Participatory democracy (also known as e-participation) involves expanding democracy beyond politics into various social areas, such as the economy, by developing a resource base that supports e-participation (including time, skills, engagement opportunities, socio-economic equality, etc.), and encouraging individuals to participate actively in the key structures of the systems they belong to. Examples include participatory digital budgeting, online citizens’ assemblies, electronic citizens’ juries, online consultations, self-managed Internet platforms (e.g., platform co-operatives), grassroots activism (cyberprotest, online petitions, etc.), and more. Forms of e-participation that relate to the relationship between governments and citizens – where citizens engage in governance through digital media – are also called open governance.

It is important to note that digital democracy does not refer to pure online activities but often involves hybrid online/offline practices. Models of democracy are not mutually exclusive but often intertwined. “Actual democratic systems can feature elements from various models of democracy and use such features to a higher or smaller degree” ( Fuchs, 2025, 88). The implication for the scenarios we develop in this white paper is that we do not create one scenario for each of the six dimensions of digital democracy. Instead, we identify two forms of digital authoritarianism where all six dimensions have been abolished and two forms of digital democracy where the six dimensions are more or less significant.

Any democracy requires a certain amount of citizen participation. Green (2010, 1069) argues that “democracy needs widespread and regular political participation from its citizens”, which is “one of the few essential criteria that unites democracies from all times and places and distinguishes them from rival forms of government”. There is a difference in the theory and practice of democracy between approaches that limit participation to the right to vote for representatives and broader understandings of participation as citizens’ involvement in governance. This means that participation can have a more “minimalist” or a more “maximalist” character ( Carpentier, 2011, 17). David Held argues that democracy can “mean some kind of popular power (a form of life in which citizens are engaged in *self*-government and *self-*regulation) or an aid to decision-making (a means to legitimate the decisions of those voted into power – ‘representatives’ - from time to time)” ( Held, 2006, 2–3). This difference has resulted in what Jürgen Habermas (1998, chapter 9) and Charles Taylor (1989) term the liberal tradition of democracy, whose main representative is John Locke, and the republican tradition, whose main advocate is Jean-Jacques Rousseau. For Habermas, the liberal model is based on the principle of the “rule of law“and the republican model on the principle of the direct “rule of the people” ( Habermas, 2026, 90). For Taylor, these two models are”incommensurable“( Taylor, 1989, 179), whereas Habermas sees the “‘equiprimordality‘of the two principles ( Habermas, 2026, 91), which is why he defines deliberative democracy as a third model that combines aspects of the two other models.

Both Locke and Rousseau stress the importance of the separation of powers in order to avoid absolute power and tyranny. Locke favours forms of representative power such as representative democracy where citizens “have a right to be distinctly represented” ( Locke 1690/1980, 82) and there is an “assembly of representatives chosen, pro tempore, by the people” ( Locke, 1690/1980, 108). Rousseau favours direct and participatory forms of democracy, arguing that sovereignty “cannot be represented for the same reason that it cannot be alienated. It consists of its essence in the general will, and the will cannot be represented” ( Rousseau, 1762/2012, 235). Therefore, he says that “the moment a people gives itself representatives, it is no longer free. It no longer exists” ( Rousseau, 1762/2012, 237).

The six models of democracy mentioned above constitute a full set of understandings of democracy. Actual democracies often combine elements from different democratic models. All these dimensions and models relate to questions of representation and participation. Representative, pluralist, and constitutional democracy align more with what Habermas and Taylor describe as the liberal model of democracy. In contrast, deliberative, participatory, and direct democracy stem from what the two political philosophers term the republican model of democracy. Therefore, when discussing the future of democracy, we simplify these six models into a distinction between representative and participatory democracy, based on the foundational division introduced by Jürgen Habermas. When discussing the future of digital democracy, scenario 3 focuses more on representative and pluralist digital democracy (representative digital democracy and society), while scenario 4 more emphasises deliberative, participatory, and direct democracy (participatory digital democracy and society). Constitutional democracy is special. Without the guarantee of the right to life, human dignity, and basic rights, there can be no democracy. The question of how to safeguard basic and human rights in online spaces is therefore important for all digital democracies, including our scenarios 3 and 4.

Robert Dahl (1998) argues that “representative government originated not as a democratic practice but as a device by which nondemocratic governments – monarchs, mainly – could lay their hands on precious revenues and other resources they wanted, particularly for fighting wars. In origin, then, representation was not democratic; it was a nondemocratic institution later grafted on to democratic theory and practice.” ( Dahl, 1998, 103). Based on Dahl, we argue that although representation is part of the democratic tradition in the form of representative democracy, it is not an inherently democratic principle. That is why we also see classical authoritarianism as a form of representation where an authoritarian leader claims to represent the people. In a similar manner, Jürgen Habermas (1962/1991) argues that monarchies are forms of “representative publicness” where the feudal powers (the monarch and the church) “represented their lordship not for but ‘before’ the people” (8). Also in dictatorships, such as our scenarios 1 and 2, there is representative power, namely the absolute power of a dictator who claims to represent the national interest of the citizens, but is unelected, so that there is no democratic representation but dictatorial representation.

### The STEEP approach

The STEEP approach can be used to analyse various societal aspects of the identified scenarios ( UN Futures Lab, 2023, 5). STEEP is a method for systematically analysing “trends, forces, and changes” in society ( Fisher *et al.*, 2020, 36). It was first introduced by Francis J. Aguilar (1967). STEEP is a systemic, macro approach that goes beyond the analysis of individuals and organisations by focusing on major societal dimensions. The macro-environmental factors it focuses on are as follows: “(1) socio-cultural factors, (2) technological factors, (3) economic factors, (4) ecological factors, and (5) political and legal factors” ( Fisher *et al.*, 2020, 37–38). STEEP stands for the initials of these dimensions. Table 2 gives an overview of these five aspects. In this paper, STEEP is used for analysing each of our four scenarios in more detail. This is because each scenario is a type of society and all societies have all of the STEEP dimensions as their subsystems. Table 2 shows example aspects of what belongs to each of the four dimensions. The examples are based on Fisher *et al.* (2020). We added further example dimensions.

**Table 2.** Dimensions of the STEEP analysis approach (further elaboration of table 6.1 in Fisher, Wisnewski, and Bakker, 2020, 6.1).

Factors	Description	Example aspects
Socio-cultural factors	Socio-cultural factors capture societies’ cultures, norms, beliefs, and behaviours, as well as demographic shifts in population distribution.	• Health care • Education (childcare, early child care, school system, universities) • Worldviews • Sports • Worldviews, religion, Ideological issues and concerns • Social relations (family life, personal life, etc.) • Lifestyle, consumer culture, and fashion trends • Population growth and segmentation • Age distribution • Media system
Technological factors	Technological factors account for technological change, including the emergence of new technologies that may disrupt a firm or industry.	• Technology maturity • Emergent technology developments • Pace of technological change • Research funding and focus • Licensing and patenting norms and regulations • Dominant technologies • Organisation of technological progress
Economic factors	Economic factors account for shifts in economic indicators and trends, and the impact of those indicators and trends on a firm and industry.	• Ownership • Working conditions • Gross domestic product growth rates • Interest rates • Employment levels • Price stability (inflation and deflation) • Currency exchange rates • Income distribution • Role of monopolies and economic concentration
Ecological factors	Ecological factors concern broad environmental issues, including the environment, global warming, and sustainable economic growth.	• Consumer preferences and demands for sustainable products and services • Environmental regulation and incentives • Access to sustainable resources (e.g., natural resources)
Political and legal factors	Political and legal factors account for the processes and actions of government and for changes in relevant laws, regulations, policies, and incentives.	• Industry laws and regulations • Political party policies and power distribution • Civil society • Public debates, public opinion, the public sphere • Citizens’ ability to influence political decisions • Voting rates and trends • Social movements • Power and focus of regulatory agencies

The dimensions of society that the STEEP method defines can also be found in social theories. For example, the systems theorist Wolfgang Hofkirchner argues that society consists of the ecosphere, the technosphere, and the sociosphere.

“The technosphere is the sphere in which means are produced, that is, in which human beings are active in innovating and applying scientific-technological tools in the course of social life. A means is a medium, in that it mediates between the starting point and the desired result, regardless of what sort of action is involved. An infrastructure of tools, methods and capabilities which comprise the overall forces of the socially living humans is the base of human systems. Technology is to augment the actors that take the role of productive forces in that they produce something when they aim at something. […] The ecosphere is the sphere in which ways are produced, that is, in which human beings work, in other words, where they use their tools, methods and capabilities to adapt nature to themselves in order to survive and construct an umwelt, where they objectify the life-support conditions of nature and appropriate nature to assure them of life support.[…] The sociosphere as a whole is the sphere in which goals are produced. It’s the sphere in which human beings perform social actions. Here they constitute what makes sense to them and realise it.” ( Hofkirchner, 2006, 15–16).

The sociosphere can be further subdivided into the economy, the political system, and culture. This distinction can be found in the works of various social theorists, such as Pierre Bourdieu (1986), Anthony Giddens (1984), and Jürgen Habermas (1985a, 1985b).

“The economy is a system in which humans in particular relations of production create use-values that satisfy human needs. […] In the political system, humans take collective decisions that govern and regulate society. Culture is the system whereby the human being is reproduced, which entails the reproduction of mind and body“( Fuchs, 2020, 55). While economy and politics have relatively clear definitions in most social theories, culture “is one of the two or three most complicated words in the English language” ( Williams, 1983, 87). While many approaches to culture define the latter as the realm of ideas that they separate from the material world, Raymond Williams argues that culture is itself material ( Williams, 1980/2020). This means that ideas have a material system they are part of. For example, ideas produced by the human brain are based on the human body. Therefore, we understand culture as the system where the human mind and body are reproduced, which implies that the health care system and sports are part of culture, just like universities and education.

In the section that follows, we outline some (but due to lack of space and time, not all) aspects of society’s six dimensions with respect to four scenarios of a potential future digital society.

## Digital futures: Four scenarios

### STEEP-S: Socio-cultural digital futures

***Scenario 1: Authoritarian digital culture – Representative digital authoritarianism’s culture.*** The regime’s overall goal in education is to instil belief in the dominant ideology and suppress critical thinking. Therefore, the government uses digital technologies as propaganda tools and means of thought control. Teachers at all levels of education must be committed members of the ruling party. Schools use an e-learning platform and e-books in all classes. The regime strictly controls and filters material published on the platform and in e-books. The state prohibits critical educational content and thus, it does not exist. Most of the content consists of the regime leader’s speeches, writings, and ideas.

The political commitment of children’s families to the regime determines educational opportunities. The regime allows only children of highly committed parents to attend university. Others must take up non-academic jobs. There is no free access to university education. In universities and research centres, scholars possess no academic freedom. The Ministry of Education strictly defines topics of research and teaching are strictly top-down. The minister of science, education, and digital affairs is also the owner and CEO of one of the country’s major tech companies. The minister channels a lot of public funding into his private pockets via the purchase of educational technologies and content.

University education and research are especially focused on STEM subjects (natural sciences, technology, engineering, and mathematics). The humanities and social sciences are seen as useless and as harbouring the danger of fostering critical thinking. Therefore, these fields were outlawed and replaced by half a semester where students focus on studying the history and main principles of the dominant ideology. The regime labels former researchers and teachers of the humanities and social sciences ‘enemies of the state’, dismisses them and sends them to labour camps. Those who complied with the new regime now work in its propaganda institutions.

Digital culture features hardware such as computers, laptops, tablets, as well as software and online platforms that are all developed by the minister of science, education, and digital affairs’ corporation. All communication is monitored, filtered, and censored.

In hospitals, doctors conduct genetic analyses of infants’ blood, which results in a genetic score. The regime considers those with a low score to be ethically inferior. The regime kills such infants. Citizens have brain interfaces and implanted medical nanorobots that scan their bodies and transmit live data on their health status to the Ministry of Health. Thereby, each individual has a health score. Those with high scores have privileges; those with low scores are punished. There is a fitness ideology that defines the ideal citizen as economically productive and militarily combat-ready. Those who do not fulfil these two ideals are publicly denounced as “parasites”. The regime deems people who object to these practices “traitors to the fatherland”.

***Scenario 2: Participatory authoritarian digital culture – Participatory digital authoritarianism’s culture.*** The regime’s goal in the realm of education is to indoctrinate children, young people, and adult learners. Digital learning is defined as participatory e-learning where the learners take an active role in selecting learning materials. However, the dominant ideology predefines and shapes the core curriculum and learning tasks. Materials that are critical of the regime and dominant ideas exist and are also present in education. However, teachers and the school system constantly frame critical thought, books, and other content negatively as “woke”. Pupils and students who support critical ideas are not punished by legal rules such as the ban from the educational system. Rather, society exerts a kind of social control where others socially shun critical people, who then become outsiders. This social control by isolation results in self-censorship and voluntary ideological submission (hegemony).

There are various e-learning platforms, digital materials, and e-books available on the market. However, the state subsidises alt-right learning platforms and publishers who focus on e-books that share the dominant ideology. Therefore, schools and universities have cheap or free access to these platforms and e-books, while ideologically independent platforms and e-books that foster critical thinking remain expensive. Market mechanisms control education. The vast majority of schools and universities adopt the ideologically aligned platforms and materials.

In universities, researchers teach freely and, inherently, select their resources without restriction, so there is formal academic freedom. However, the state directs public funding to research projects and researchers whose academic knowledge aligns with the dominant regime. Other research and teaching are tolerated but are much less financially viable due to a lack of funding, have low visibility, and little impact on society. The social sciences and humanities exist in many universities, but the regime deliberately keeps their share of the overall budget extremely low.

In health care, there are private/public partnerships between the state and some large health care corporations that control the pharmaceutical market and operate large hospitals. The owners of these corporations are ideologically aligned with the ruling party. There are also other smaller pharmaceutical companies and health care organisations that, however, hardly receive public funding. In medical research, the state provides public funding especially for corporate research undertaken by biotechnological corporations whose owners. Other medical research is not prohibited but receives little to no public funding.

A digital platform crowdsources medical care work to volunteers. Such work includes, for example, elderly care, childcare, palliative care, or supportive care. Ideologically, the regime presents this outsourcing of health and care work from paid care and medical employees to unpaid citizens as the democratisation and communalisation of the health and care system via digital technologies that enable participatory care. The government presents this participatory care initiative as a new form for strengthening social cohesion and local community ties. The ideological conviction that the state should, to a large degree, privatise healthcare drives the actual motivation.

***Scenario 3: Representative digital culture - Representative digital democracy and society’s culture.*** The educational system pursues a dual goal: it should educate learners for the job market and enable them to become reflective and responsible citizens. Pupils and students use traditional and digital means of learning – printed books and e-books, pens, pencils, and paper, as well as tablets, learning apps, learning games, and e-learning platforms. Either entire cities or schools decide how they use parts of their budgets to give their students and teachers access to electronic materials and technologies. There is a market for educational technologies where many companies and organisations are active, but that is dominated by a smaller number of private companies. Non-commercial and FLOSS (free, libre and open-source software) e-learning platforms and publishing experiments exist but occupy a marginal position.

There is freedom of academic teaching and research. However, STEM fields receive a much larger share of funding and budgets than the social sciences and humanities. In universities, digital technologies are widely used for e-learning and the publishing of books and articles (e-publishing). There is a rivalry between private for-profit publishers and software developers in the higher education market and not-for-profit initiatives, journals, publishers, and tech providers (e.g. FLOSS software development projects such as Moodle), where the former dominate.

The electronic patient record and health IDs constitute the major digital project in the healthcare sector. The goal is to improve the flow of information between health care providers in order to improve patient care, optimise diagnostics, and reduce administrative and medical costs. AI analyses the electronic patient record and provides recommendations to citizens based on their medical records. Health care providers foster gamification via a health gaming platform where users obtain benefits for documenting that they engage in sports, eat healthily, and practice a healthy and sustainable lifestyle.

***Scenario 4: Participatory digital culture – Participatory digital democracy and society’s culture.*** The main goal of the educational system is to empower democratic citizenship and learners’ capacities to think and act critically. Schools and universities have curricula with defined goals. The system encourages pupils and students encouraged to participate in selecting learning methods. Learning is more flexible in terms of pupils’ and students’ individual choices. Schools are organised in a more participatory manner, with key decisions taken by co-determination bodies comprising learners, parents, and teachers. For example, they elect the school’s management.

Nurseries, schools, and universities are adequately and publicly funded; therefore, there is no shortage of resources and personnel. As a consequence, teachers can provide sufficient support for learners. Schools and universities experiment with digital participatory budgeting, in which educational institutions allocate a share of the budget to projects suggested by learners. Thereby, students and pupils practice democracy as part of their everyday learning environment.

Schools regularly use e-books and e-learning platforms. Non-commercial and not-for-profit models dominate the educational media and technologies. In this context, FLOSS (free, libre and open-source software) e-learning platforms and software, not-for-profit educational and academic open-access publishers (so-called diamond open access), as well as educational platform co-operatives play an important role. Supported by digital technologies, learning has become more participatory and more social, so that pupils and students are encouraged to work more in teams that produce content for learning purposes. Schools experiment with sharing such content with others so that they encourage peer engagement.

Educational institutions like universities maintain freedom of academic teaching and research. In universities, the social sciences and humanities now receive a greater amount of funding and academic boards integrate them into other degree programmes. This means that all students, including those studying STEM subjects, now have the opportunity to learn about society, democracy, sustainable development, (in) justice, philosophy, and history. Thereby, the STEM subjects develop a greater awareness of societal problems.

Universities use digital technologies mainly for e-learning and e-publishing of books and articles. Digital education resources, such as e-learning platforms, e-books, journals, databases, and software, are not-for-profit and open source by default. Not-for-profit diamond open-access publishers are the dominant actors in academic publishing. The use of FLOSS software now carries more importance in universities than reliance on expensive proprietary software licences.

Health care is now organised around the principle of patient-centred care, allowing doctors to take adequate time to discuss health issues and treatment options with patients and their close ones. This system empowers patients to become informed participants in conversations with doctors and decisions concerning their health, while recognising that doctors are the experts. There are electronic patient records and health IDs to improve the flow of information between health care providers, make health information available to patients, and support the conversations between patients and doctors. The digital patient record includes a function that allows patients to record observations about their well-being and to comment on the entries that doctors make. Overall, a more deliberative and participatory structure defines the health-care system than in former times. This does not mean that all patients become their own doctors, but that there is a more patient-focused health care system and more informed discussion between doctors and patients.

### STEEP-T: Future digital technologies

All scenarios have to do with future uses of technologies. Here is a list of ten types of technologies that we see as defining the future technological landscape:

1. Environmental technologies:

Technologies that humans use in order to use natural resources in particular ways, energy generation, food production, agriculture and farming, water and waste management, technologies for environmental sustainability, digital infrastructures, green computing, etc.

2. Health technologies:

Wearable technologies, technological implants, genetic and bio technologies, medication to diagnose, treat, prevent, or relieve symptoms of diseases and health conditions, surgical techniques and machines, care technologies, reproductive technologies, palliative technologies, robotic and minimally invasive surgery, Artificial Intelligence for diagnostics and treatment, personalised medicine through genomics, telemedicine and remote patient monitoring via wearable devices, 3D bioprinting for tissue, virtual medicine etc.

3. Social media and streaming platforms:

User-generated content, entertainment, and culture platforms (music, film, audio-visual content, podcasts, etc.), video platforms, live streaming platforms, messaging technologies, wikis, microblogs, video and photo sharing platforms, e-learning technologies, etc.

4. News and journalistic technologies:

Newspapers, magazines, newsletter platforms (Substack, Medium, Table Media, etc.), investigative journalism technologies: OSINT (open source intelligence), fact-checking technologies, news aggregators (Ground News, The European Correspondent), algorithmic news aggregation (Google News, Ground News), expert-based news aggregation (The European Correspondent,, community-driven news and information aggregation (Digg, Reddit), etc.

5. Artificial intelligence and automation technologies:

Generative AI, AI assistants, smart technologies, virtual and augmented reality technologies, robots, drones, self-driving vehicles, natural language processing (NLP) technologies, etc.

6. Political information technologies:

News and journalistic technologies, political information technologies, political communication and debate technologies, technologies of civic engagement, digital activism, e-participation technologies, civic tech, open governance technologies, online deliberation technologies, social scoring technologies, security and surveillance technologies, etc.

7. Mobility technologies:

Public transport technologies, personal transport technologies, space technologies, logistics, delivery technologies, drones: unmanned aerial vehicles (UAVs) & unmanned maritime vehicles (UMVs), drone swarms, self-driving vehicles, urban air mobility (UAM), micromobility (e-scooter, e-bikes, future of the bicycle, etc.), streets and transport ways, smart city technologies, space travel technologies, life on other planets, terraforming technologies, etc.

8. Smart city and home technologies:

Smart home, smart city, smart energy management, intelligent traffic systems and management, digital urban planning, urban green spaces, urban security and surveillance technologies, urban social scoring technologies, digital village technologies, global village technologies, etc.

9. Data technologies:

Big data, small data, computational social science, data analytics, digital humanities, digitalisation projects, personalisation technologies, public service algorithm, data and server farms, cloud data platforms, Internet of Things, etc.

10. Computing hardware and software:

Quantum computing, open-source software, free software, proprietary software, semiconductors, smart phones, smart screens (tablets, television, etc.), GPUs and CPUs, standards, materials, future computing, etc.

***Scenario 1: Authoritarian digital technology – Representative digital authoritarianism’s technology.*** In scenario 1, there is a strong focus on the development of surveillance and control technologies, big data technologies, and dataveillance (big data-based surveillance). Privacy-enhancing technologies have been prohibited by law, and all data protection legislation has been abolished. Big tech corporations control research and technology development. The digital giants’ CEOs and owners are not just managers but also politicians who are committed to the ruling regime’s ideology. Three big tech companies dominate research and development. The owner of company one is also the minister of science, education and digital affairs. The owner of corporation two is the Minister of Security and Defence. The owner of corporation three is the Minister of Economic, Cultural, Social, and Financial Affairs. Corporation 1 controls the educational sector, where all nurseries, schools, universities, and research organisations are part of one big company. Corporation 2 holds a monopoly in the arms, security and defence industries. Corporation 3 is the only company in the e-commerce, media, entertainment, cultural, finance, manufacturing, and health industries. There is a close alignment between monopolist corporations and the state. Society is a state monopolist (digital) capitalism. The dominant companies are government-aligned corporations, which means that the owners of the dominant corporations play key roles in the government that rules in a dictatorial manner.

***Scenario 2: Participatory authoritarian digital technology – Participatory digital authoritarianism’s technology.*** In scenario 2, there is a focus on the development of a wide variety of digital technologies with a focus on entertainment and consumer technologies, as well as citizen engagement technologies. Public/private partnership drive all technology development. The state is a multi-party system with governance mechanisms that privilege the power of the ruling party, which uses digital technologies to cement its power and weaken the opposition. There is a creeping form of fascism. The economy consists of private for-profit companies as well as state-controlled companies that control some key critical infrastructures. The ruling party’s key functionaries and the dominant corporations’ CEOs are ideologically aligned and cooperate in the form of public/private partnerships, especially in the development and organisation of (fake) news media, digital platforms, e-voting and e-participation technologies, and (dis)information. Together, they play a key role in the manipulation of public opinion.

***Scenario 3: Representative digital technology – Representative digital democracy and society’s technology.*** In scenario 3, there is a focus on developing a wide variety of digital technologies that are regulated by laws focused on privacy, data, and consumer protection. The goal is to make a large range of digital public services and digital consumer services available to everyone. There is a dual digital economy. On the one hand, large for-profit tech companies are developing and operating digital consumer services. On the other hand, new publicly owned digital companies have emerged that focus on the development of digital public services. Digital public services aim at fully digitalising representative democracy’s institutions, such as parliaments, elections, public services, municipal administration, health care, education, etc. Authorities use digital technologies to open up governance to a variety of stakeholders, including citizens, civil society, and industry, making representative democracy more based on citizens’ inputs and the co-creation of public policies and services.

***Scenario 4: Participatory digital technology – Participatory digital democracy and society’s.*** In scenario 4, there is a focus on developing a wide variety of privacy-enhancing, democratic, environmental, and societally sustainable and human-centred digital technologies. In the digital economy, we find for-profit digital corporations, platforms and digital co-operatives that are not-for-profit organisations managed and controlled by their workers and users, and public service media organisations that are independent from the state, profit interests, and ideologies. Not-for-profit public and commons-based organisations together play an important role in the digital economy. There are public/commons partnerships where organisations such as Wikipedia and public service media such as the BBC and ARD co-operate in the development and provision of digital services and platforms. There is a strong focus on developing e-participation technologies that enable meaningful citizen participation in matters of public concern. Participation goes beyond trivial matters such as what colour park benches should have. Some structures enable citizen participation. E-participation routinely takes on hybrid online/offline forms.

### STEEP-E: Digital economic futures

***Scenario 1: Authoritarian digital economy – Representative digital authoritarianism’s economy.*** This scenario features an unregulated capitalist economy. The majority of economic resources are privately owned, except for critical infrastructure, which is state-owned. Three large corporations centralise and monopolise markets. All other companies in the country are subsidiaries of one of these corporations, forming a network of subsidiaries. The three owners of these corporations are part of the ruling regime. The ruling government exempts their companies from corporate taxes. The owner of the corporation holding a monopoly in the realm of the educational sector is the Minister of Science, Education and Digital Affairs. The owner of the second monopoly company that controls the arms, security and defence industries is the Minister of Security and Defence. The owner of the third monopoly company, which controls the e-commerce, media, entertainment, cultural, finance, manufacturing, and health industries and is the largest national employer, is the minister of economic, cultural, social and financial affairs. This ministry also oversees all labour affairs.

The regime has weaponised the social security system to guarantee loyalty: Only members of the ruling party have access to state-provided healthcare, unemployment benefits, pensions and other social insurance schemes. Alternative forms of ownership, such as cooperatives, have been prohibited, as have labour organisation and collective bargaining via unions and worker councils. Any independent trade union is illegal, and the only existing “union” is a state-controlled entity designed solely to enforce productivity quotas. Citizens are dependent on the state or the three corporations and their subsidiaries for employment; therefore, to survive, people must align themselves ideologically with the regime.

In this scenario, society is a fascist state monopoly capitalism where there is a close alignment between state power and corporate power. Both the state and the economy are ruled by a political-economic elite in a fascist manner. The ruling party has declared that class relations no longer exist because everyone is united by pride in nationhood. Nationalism ideologically distracts attention from the existence of deep class divisions, where severe wealth disparities exist between a small elite who control the economy and the majority of people who are forced to live in poverty if they do not join the ruling party. There is no minimum wage regulation due to the regime’s strict anti-migration policies. A severe labour shortage exists due to the regime’s anti-immigration policy, which is why the regime has increased the standard working week from 40 to 50 hours without a wage increase.

Just like political protests, strikes, and unionising have been outlawed. Attempts to collectively organise labour are severely punished. Authorities highly control and monitor labour. Big data systems are used primarily as a control mechanism, including close digital surveillance of workers. The state uses artificial intelligence to predict dissenting behaviour and to counter it through ideological manipulation and criminal prosecution directly. Forms of repression and punishment include withdrawal of access to social security and deportation to labour camps for ideological re-education and brainwashing. There is the death penalty for attempts to organise strikes or unionise workers. The working conditions for the general masses in these big corporations are precarious. Those who are not party members suffer from low wages and are stripped of worker rights. Only party members benefit from a sufficient salary and other benefits.

In the digital economy, algorithms, software, and other digital content (music, videos, animations, images, etc.) are proprietary and controlled by the big three digital corporations. These three capitalist corporations together control and own all Internet platforms. There are no alternative platform models to digital capitalism. The latter is controlled solely by three digital corporations that are also fully politically aligned with the regime.

The platform economy has become the primary avenue for service provision. Retail and on-location businesses have been abolished; therefore, groceries and products for general use must be ordered via online platforms that are directly and indirectly owned by the three corporations. This leads to the state having access to the buying and consumption patterns of citizens since the corporations are obliged to share such data with the main ministries (the Ministry of Science, Education, and Digital Affairs, the Ministry of Security and Defence, and the Ministry of Economic, Cultural, Social, and Financial Affairs). These then analyse the given data to enforce a kind of social scoring system. This scoring system is central to the regime’s monitoring and surveillance efforts. The social scoring system works as a baseline for a state-mandated app, the manufacturer preinstalls on every device the citizens use and the system automatically connects to their social security number. Citizens are forced to use this one app, as the app is the only avenue to participate in society. It integrates payments, social media, transportation, food orders, health services and other government services.

Society is highly unequal in terms of income and wealth distribution. The use of robotics and AI for large-scale labour automation has further worsened this inequality, affecting not just the manufacturing section but also highly qualified professional labour. This has led, on the one hand, to growing unemployment and, on the other hand, to the extension of precarious routine jobs. This leads to top-down social stratification, as artificial intelligence serves as a barrier. This barrier widens the gap between the rich and the poor and keeps a large part of the population trapped in precarious working conditions.

Universities, education, arts and culture, media, and journalism are important intellectual realms of society where new ideas and political resistance have often originated. Therefore, authoritarian regimes have often exerted special forms of repression and control in these sectors. In our scenario, the regime uses generative AI for large-scale automation of workers in these intellectual sectors; consequently generative AI systems produce most of the books, artworks, news, movies, songs, video games, advertisements, podcasts, and other cultural products. AI culture is a propaganda tool because the regime’s engineers program the AI systems to produce only culture that propagates the regime’s ideology, alongside depoliticised tabloid entertainment. The workforce in cultural companies, such as news production, consists, on the one hand, of AI-driven state-controlled robot workers (such as AI journalists), and on the other hand, of highly loyal party members who have proven that they support the regime’s ideology.

***Scenario 2: Participatory authoritarian digital economy – Participatory digital authoritarianism’s economy.*** In this scenario, the state merely provides the framework for economic activity, keeping regulation to a minimum. Too much regulation in labour, consumer, and environmental protection, as well as oversight of financial transactions and progressive taxation, is said to undermine economic growth. This lack of adequate regulation has led to monopolisation and a high level of ownership concentration in the economy. The state does not outlaw small businesses and co-operatives (self-managed companies that are owned and managed by those who work in them), but they cannot compete with large corporations, and therefore bankruptcy or mergers and acquisitions repeatedly force them out of the market.

The ruling party maintains a transactional relationship with the few private corporations that dominate the economy because it is financially dependent on them. In return, the government implements economic policies that benefit the corporations and the wealthy. The state taxes income at a flat rate of 15%, which benefits the wealthy and has deepened the gap between the rich and the poor. Company profits are taxed at a rate of 5 per cent for small and medium enterprises. The government exempts large corporations from corporation tax, thereby increasing the centralisation of the economy. There is a minimum wage, which is, however, only slightly above the poverty line. The ruling party is strictly anti-immigration. Its policies in this regard have led to a severe labour shortage, which the government has attempted to address by raising the standard working week from 40 to 45 hours without increased pay.

There are both private and public services across social care, healthcare, education, and public transport. The state often underfunds public services. This means that many citizens have to rely on private insurance to cover their financial well-being and retirement benefits, as they are dependent on private companies for social security. On the other hand, the government publicly subsidises private insurance and service companies.

There is freedom of association, including the freedom to form trade unions. However, the media often defames trade unions. As a consequence, the idea of unionisation is rather unpopular. Corporate employers operate their own healthcare funds, schools, grocery stores, transport services, and apartment complexes, which employees can access as long as they comply with the working conditions. These services are better funded and maintained than government-operated services and are therefore seen as the better alternative.

In the digital economy, algorithms, software, and other digital content (music, videos, animations, images, etc.) are predominantly proprietary. Large digital corporations control such resources. Creative Commons and other non-commercial forms of digital organisation exist but remain a niche phenomenon because the state subsidises the big digital corporations. As a consequence, the corporate digital giants dominate the Internet. Three big tech corporations own and operate ninety-five per cent of all Internet infrastructure and platforms. There are alternative models, such as platform co-operatives, but they are small, rather unknown, and not widely used.

The economic infrastructure relies on gamification and nudging. Gamification works to ensure regime-friendly participation by managing and coercing economic behaviour that benefits the state. There is a social scoring system operated via apps. Participation is voluntary. Citizens document various behaviours via the app, and the regime assesses their behaviour positively, granting scores for regime friendly behaviour. There are no negative scores. Those with high scores receive benefits that others do not obtain. The regime uses artificial intelligence to assess the documented forms of behaviour. It is designed to nudge citizens toward behaviour and choices sanctioned by the state. Citizens are nudged to perform activities and other forms of unpaid labour to be rewarded and to maintain or increase their social score.

Consuming goods from state-loyal/aligned brands boosts the social score, and buying from unapproved outlets lowers it. A higher score grants citizens privileges such as access to better jobs, lower loan interest rates, cheaper housing, lower grocery prices, and faster public services. The result is that many citizens engage in labour and activities that the government desires without using physical violence to bring about this behaviour. Rather, digital gamification is used as a subtle form of social coercion to incentivise citizens to engage in regime-friendly behaviour and avoid political activities such as speaking out against the government, the autocratic societal system, engaging in protests, etc. The social scoring system increases inequalities. Citizens with higher scores enjoy better living conditions and are held in high regard. Society views them as having earned their wealth in the proper way. Consequently, citizens with a lower score are held responsible for their own poverty and social downfall. This further encourages state-friendly behaviour by promoting an illusion of attainable wealth, which the system directly couples with the desired conformist patterns.

The state does not centrally control generative AI systems. Everyone can use such systems. The dominant news and entertainment corporations share the ruling party’s ideology, which is why the dominant generative AI systems are designed to create hyper-personalised propaganda to nudge citizens into greater alignment with the prevailing political regime.

Robotics and artificial intelligence are organised through the market, so companies are free to use or not use them as tools of automation. The manufacturing sector is widely automated. In the realms of professional knowledge work, which requires higher education, some companies make large-scale use of generative AI systems to create news, books, artworks, movies, songs, video games, advertisements, podcasts, and other cultural products and to replace cultural workers. In contrast, others merely use generative AI to augment the labour of professional workers. Highly automated media and culture companies tend to align with the government’s ideology. Their labour costs are lower than those of more labour-intensive cultural companies that use AI to merely augment and not automate professional knowledge work. The highly automated cultural companies also have higher productivity than others. As a consequence, they dominate the cultural markets. The news and information content they produce is strongly supportive of the government. In addition, there are lots of tabloid entertainment programmes. The more labour-intensive media and cultural companies produce high-quality news, educational content, and culture. However, because these companies do not dominate the market, their content reaches only a small share of the population, while the majority ignore it.

***Scenario 3: Representative digital economy – Representative digital democracy and society’s economy.*** In this scenario, the state acts as a mediator between private business and labour organisations. It regulates the economy through measures that define working conditions, workplace security, minimum wages, working time standards, consumer protection, environmental protection, etc. The state provides a legal framework for representatives of capital and labour to negotiate wage increases and working conditions.

Unions and worker councils are essential parts of the economy. Furthermore, worker co-operatives play a certain role in the economy as an alternative form of ownership. There is a mixed economy where private for-profit companies employ 60 per cent of the workforce, the public sector employs 20 per cent, the non-profit third sector employs 10 per cent, and worker co-operatives employ 10 per cent. The state supports self-management by financially subsidising the creation of cooperatives and labour-managed firms. This state support takes the form of a special legal status for co-operatives and third-sector organisations. Co-operatives pay slightly lower corporation tax than for-profit companies. They do not pay corporation tax insofar as they have a defined public purpose. Examples are charities or organisations that aim to advance the social good. Such legal measures are seen as a long-term effort to strengthen democracy by expanding it to the economic sphere.

The state is a classical welfare state that allocates a significant proportion of its annual budget to public and social security services, particularly health service infrastructure, unemployment benefits, pension and retirement funds, universal childcare, state schools, public transport, and housing subsidies for low-income households. This is made possible by progressive taxation of wealth and income. The top marginal income tax rate is 60 per cent. It is applied to income above 500,000 Euros. The government strictly regulates and taxes financial transactions at a higher rate than income from labour. The government views a high degree of wealth and income inequality as a threat to democracy. It has therefore pledged to reduce the wage gap between the highest and lowest incomes in all industries to a ratio of 1:20 within the next 20 years. Furthermore, it provides every citizen with a starting stipend of 50,000 euros upon turning 18 and helps school and university graduates set up socially responsible small businesses and cooperatives. This is part of the state’s initiative to promote and support social entrepreneurship and financial independence.

A regulated digital capitalism defines the digital economy. In this economy, algorithms, software, and other digital content (music, videos, animations, images, etc.) take on both proprietary and common forms. The proprietary digital economy dominates, but the digital commons play an important role. In terms of ownership, the digital economy therefore consists of private for-profit digital companies, not-for-profit public digital service companies, and not-for-profit platform and digital co-operatives. The second type of digital company operates in niches such as public companies in e-voting, e-government, e-health, and other digital public services. Platform co-operatives are especially active in certain creative and service industries (open-access publishing houses, image-sharing platforms, music streaming, freelance platforms, ride-hailing platforms, food-delivery platforms). In such industries, worker ownership aims to increase wages relative to classical for-profit platform corporations (e.g. Uber, Deliveroo, Fiverr, Upwork, TaskRabbit, Guru, Shutterstock, Spotify, etc.), which retain a significant share of each transaction and turn those commission fees into profits.

The state regulates the digital economy in the form of privacy and data protection legislation, minimum wages for platform workers agreed upon at the transnational level, the legal support of the creation of (digital) labour unions, the closing of tax loopholes that in former time enables transnational digital corporations to avoid paying corporation tax, the enforcement of anti-trust laws, requiring platforms to remove illegal content immediately and to explain to users how the utilised algorithms work, etc.

Digital technologies have two major applications: public administration and private businesses. The state develops its own digital technologies for digital public services and open governance. Thereby, there is a public professional digital media workforce that involves researchers, software engineers, designers, etc. The most popular digital public services are the electronic tax return, the electronic health card, and the electronic town hall, an app that enables citizens to directly communicate with elected representatives and government and public administration to provide information to citizens.

The development and use of artificial intelligence, robotics, big data, and ever newer digital technologies are, on the one hand, market-driven and on the other hand state-regulated. Given that the economy develops faster than legislation, the state’s regulatory capacities tend to lag behind technological innovations. New technologies tend to remain unregulated for some time after their emergence and diffusion.

Companies use robotics and artificial intelligence to automate processes. Digital automation not only affects manufacturing and low-skilled service jobs but has also, with the diffusion of generative AI into the economy, affected high-skilled professional knowledge work. The state’s Automation Act regulates digital automation. It requires companies that introduce an automated system or robotic system that is expected to make ten or more jobs superfluous or that affects more than ten per cent of the total workforce to carry out an Automation Impact Assessment (AIA) 120 days before the introduction of that system. The assessment is submitted to the Department of Labour, which can request amendments to the planned use up to 45 days before the start. Employees, work councils, and unions can submit opinions to the Department about the effects the new system could have. The department considers such submissions alongside the AIA. In cases of concern, the Department of Labour often acts as a conflict arbitrator, mediating the resolution process between representatives of both the employer and employees.

***Scenario 4: Participatory digital economy – Participatory digital democracy and society’s economy.*** In this scenario, democracy is not limited to the formal political system but extended to the economy. The result is a mixed economy consisting of different models, including worker co-operatives, not-for-profit third sector organisations, public service companies, as well as private for-profit companies. Co-operatives account for 30 per cent of all employees, third sector organisations for 20 per cent, public service companies for 30 per cent, and private companies for 20 per cent. This means that the not-for-profit sector is dominant.

In co-operatives, decisions are made co-operatively so that workers are at the same time decision-makers and managers who elect management for operational matters. Public service organisations tend to be large, which requires the delegation of decision-making power. The workforce elects managers for specific terms. In third sector organisations and private companies, elected work councils play an important role and are part of the executive board, where they represent worker interests. In economic partnerships, the dominance of private/public partnerships has been replaced by public/commons partnerships involving state companies and not-for-profit organisations.

The state plays an active part in the economy as an employer in the public sector economy, regulating markets, providing the legal framework for economic democracy, managing economic and financial crises, and legally defining overall goals such as the UN Sustainable Development Goals. The economy’s primary goal is to satisfy human needs rather than to maximise private profits. The management of societal resources, such as labour and means of production, is organised and determined by members of society via democratic institutions.

State legislation defines a minimum wage that guarantees everyone a decent life. Housing market prices are regulated, and housing involves a significant degree of affordable council homes (flats and houses) and housing co-operatives, which is a regulatory measure against exploding housing prices. Thanks to increased productivity, the working week has been reduced from 40 to 32 hours, with full wage compensation, and the long-term goal of reducing it even further. Tax and wage legislation has resulted in a significant reduction in poverty and socio-economic inequalities, which, in turn, has had a positive effect on crime. The state makes large investments in public education, which has doubled the share of the population that has completed a university degree. The increase in available time, educational levels, and equality has had positive effects on democracy, as more citizens now regularly engage in public political debates, civil society action, and participatory democratic politics such as participatory budgeting. Democracy has thereby become stronger and more dynamic.

In the digital economy, algorithms, software, and other digital content (digital music, videos, animations, images, etc.) are predominantly licensed under Creative Commons and open-source licenses. The digital commons dominate over proprietary digital content. The digital economy consists of for-profit digital corporations, platforms and digital co-operatives that are not-for-profit organisations managed and controlled by their workers and users, and public service media and Internet organisations that are independent from the state, profit interests, and ideologies. Not-for-profit digital organisations dedicated to advancing the digital public sphere and digital democracy have successfully challenged the monopoly power of US and Chinese digital giants such as Alphabet (Google and YouTube), Meta (Instagram, Facebook, and WhatsApp), ByteDance (TikTok), Amazon, and Apple. Public service Internet platforms have emerged as a global co-operation of public service media organisation such as BBC, ARD, ZDF, PBS, France Télévisions, RTVE, RAI, etc. which form a global consortium that has developed successful Internet platforms such as a public service online video sharing and streaming platform that competes with Alphabet’s YouTube and commercial video and television streaming platforms such as Netflix, Amazon Prime, and Disney+. Public service Internet platforms are especially active in the audiovisual sector, while platform co-operatives dominate services that process lots of personal data, such as instant messaging, social networking, online shopping, locational services, online search, etc. This means that public service Internet platforms have challenged the power and dominance of YouTube, Netflix, Amazon Prime, Disney+, and Spotify; and platform co-operatives have challenged the power and dominance of Instagram, Facebook, TikTok, Amazon, Google, Uber, Deliveroo, and WhatsApp. The corporate digital giants continue to exist, but in many countries, they are no longer the dominant platforms. In the realm of hardware production where commercial actors continue to play an important role, public/cooperative partnerships have resulted in successful joint ventures that manufacture computing hardware such as mobile phones, laptops, desktop computers, consoles, printers, etc. that is energy-efficient, environmentally sustainable, upgradeable and reuseable (green computing), and developed and assembled by workers who are paid a fair wage.

Robotics has been used to automate many forms of menial labour. For example, the jobs of toilet cleaners, refuse collectors, and sewer and wastewater divers have disappeared. Such waste management labour is carried out by robots under human supervision. There are upskilling programmes so that those who lose their jobs do not become unemployed but instead undergo paid reskilling into higher-qualification jobs, so that they normally earn higher wages than before. This has given workers who previously performed such labour the opportunity to pursue advanced education in the humanities, social sciences and natural sciences. As a result, technological and scientific advancements are more widespread, since deep engagement in intellectual activities is no longer the privilege of those with sufficient resources.

In the realm of care labour, robots are used to carry out physical labour such as carrying people and objects. A societal agreement and legislation ensure that society does not automate the social dimension of care and educational work. This means that there are no robotic nurses, medical doctors, teachers, psychotherapists, childcare workers, elderly care workers, palliative care workers, etc. Educational and care workers are well paid and have lower standard working hours than others, which incentivises more people to enter and remain in these professions. Educational and care workers make use of AI systems and professional social networks to obtain scientific information and advice on specific care questions.

Generative AI has the capacity to foster large-scale automation of highly-qualified knowledge workers such as journalists, teachers, researchers, judges, lawyers, architects, designers, artists, consultants, media and entertainment workers (singers, actors and actresses, theatre performers, film directors, screenwriters, novelists, voice actors, photographers, composers, etc.) medical doctors, psychotherapists, etc. The reason is that generative AI can simulate highly qualified knowledge work, including tasks that require a high level of inventiveness and creativity. In our scenario, society has actively chosen not to automate highly skilled jobs by generative AI-driven systems, intelligent robots, or other systems. The reason is that robots and AI systems do not have emotions, feelings, intelligence, judgment, morality, ethics, self-consciousness, empathy, compassion, semantics, understanding, meaning-making capacity, intentions, or the capacity to anticipate the future. They act merely based on software programmes, algorithms, and databases. AI agents and robots are fully instrumental and have a technological logic. In our scenario, a law prohibits the automation of such jobs. In professional knowledge work, workers and companies use AI to augment and support, but not to replace, creative knowledge labour. Knowledge workers utilise AI systems as support and augmentation of their work, but reject the idea that their jobs can and should be fully automated.

### STEEP-E: Digital ecological futures

In 2023, there were the following sources and shares of world electricity production: nuclear (9%), fossil fuels (60%), renewable energy (31%). Fossil fuels involved coal that accounted for 34% of world electricity production, natural gas (22%), and oil (3%). Renewable energy included hydroelectricity (15% of world energy production), wind energy (8%), solar energy (5.5%), and biomass and waste (2.5%). ( EIA, 2025).

Planned obsolescence and hardware companies’ branding strategies generate lots of e-waste. In 2014, there were on average 6.4 kg of annual e-waste per capita of the world population; this amount rose to 7.3 kg per capita in 2019 and 7.8 kg in 2022; it is projected that it will further increase to 9 kg per capita in 2030 ( Baldé *et al.*, 2024). Running the Internet and data networks consumes about 4–8% of world energy production ( De Decker, 2015 & Malmodin *et al.*, 2023).

***Scenario 1: Authoritarian digital ecology – Representative digital authoritarianism’s ecology.*** In this scenario, there is a big focus on fossil fuel generation and use. The ruling regime sees climate change as a hoax and something natural that societal development and industrialism have not caused. It supports the oil and gas industry, which dominates energy production and is controlled by one big private corporation. Nuclear energy is also pivotal to the regime, and the nuclear industry is solely controlled by another big private corporation. The fossil industry provides 80 per cent of the national electricity production. The coal industry accounts for 30 per cent of electricity production, oil for 30 per cent as well, and gas for 20 per cent. The nuclear power industry produces 20 per cent of electricity.

The regime views renewable energy sources as part of a “green ideology” that it rejects. Therefore, the state outlawed renewable energy production and dissolved all green energy companies. All wind turbines were dismantled with the argument being that they disfigure the environment. The death penalty was introduced to counter the promotion of green energy production as well as attempts to privately create such energy sources. As a consequence of these policies, climate change further intensifies, along with it the frequency of natural disasters such as tsunamis, hurricanes, flooding, extreme heat waves, water scarcity, etc. The privileged elite and party members are living in zones less affected by climate change. Regime critics and scapegoated minorities are left in or deported to high-risk areas where there is flooding, natural disasters, etc. where many of them die. This is an ecological form of getting rid of the regime’s opponents.

The assertions by political opponents that fossil fuels have a definitive expiration date are dismissed by the ruling regime as fake news. Renewable energy sources continue to be delegitimised as purely ideologically driven, while fossil fuels are promoted and subsidised under the guise of national tradition. Although the regime is internally aware that current resources are finite, it maintains a lack of transparency, categorising any reports to the contrary as misinformation. Simultaneously, a gradual strategic pivot toward expanded nuclear energy is underway.

By strategically centring the national discourse on nuclear energy, the regime further delegitimises renewable alternatives. This shift serves a dual purpose: it functions as both a rhetorical justification and empirical proof for the regime’s platform, effectively providing the necessary leverage to silence or marginalise political dissidents.

There is no awareness of the problem of e-waste. Hardware companies and the regime promote excessive consumerism, resulting in very short lifespans for hardware such as computers, mobile phones, and tablets. In our scenario, the average annual e-waste per capita amounts to 50 kg. Attempts to create green computing devices with reusable hardware components were abolished and outlawed.

Given that the regime in this scenario is a highly repressive and militarised surveillance society where massive amounts of personal data are generated, analysed for identifying enemies of the state, and forever stored in data centres, there is a massive amount of fossil and nuclear energy that digital networks consume. They account for 35 per cent of the overall electricity consumption.

In the realm of transport, public transport was abolished because the regime is hostile towards anything based on public ownership. The society in our scenario is car-centred. The private car is the main means of transport. There are also privately owned self-driving taxis that one can rent.

***Scenario 2: Participatory authoritarian digital ecology – Participatory digital authoritarianism’s ecology.*** In this scenario, there is a variety of energy sources, including nuclear, fossil, and renewable energy. The regime organises a digital plebiscite regarding what energy future should be realised. A digital plebiscite is an instant digitally-mediated referendum with yes/no answers. It favours the combination of fossil fuel plus nuclear energy with the goal of reducing fossil energy consumption and heavily increasing nuclear energy, which is why it provides public funding to nuclear energy companies. Because of public funding, the prices of nuclear energy are low, while renewable energy is very expensive.

The option of increasing reliance on nuclear energy wins the plebiscite. The risk of nuclear disasters intensifies. Green energies are not rejected but not seen as the best solution.

E-waste continues to be a major environmental problem that is connected to the unsustainable character of the digital economy, which is, like today, dominated by large transnational US and Chinese hardware and software corporations. Green computing and electric cars are promoted as far as they enable new possibilities for corporations’ profit generation as part of green capitalism. Green capitalism based on renewable for-profit energy exists alongside hyper-industrial fossil capitalism based on fossil for-profit energy.

The government does not deny that climate change exists or that it is a societal problem. It propagates technical measures such as carbon dioxide removal (CDR) and solar radiation management (SRM) as the two main methods for tackling the climate crisis. Private businesses seeking to yield profit organise such technologies. Furthermore, it uses the emissions trading system (cap and trade scheme) as a way to disincentivise limitless fossil fuel use and emissions.

In the realm of transport, private companies operate public means of transport, such as trains, buses, and tramways, in big cities with relatively high prices. The car is an important means of transport, especially in rural areas where there continues to be a lack of public means of transport, in order to save investment costs.

***Scenario 3: Representative digital ecology – Representative digital democracy and society’s ecology.*** In this scenario, there is a variety of energy sources. Climate change is acknowledged as a major societal problem that requires countermeasures to reduce fossil fuel and energy production. The state provides public funding to green energy production, which is primarily a domain operated by private companies. Researchers conduct experiments with new forms of the environmental economy, such as green energy co-operatives. The state is one of the actors in renewable energy production. There is a small number of public companies in the energy sector that are focused on pushing solar and wind power. To do so, the government brings a certain amount of land into public ownership via eminent domain, to be used for wind turbines and solar panels operated by public energy companies.

In electricity production, renewable energy is dominant and accounts for 60 per cent of all generated electricity. Nuclear energy accounts for 25 per cent of the electricity the country produces. There are investments and research endeavours into trying to make nuclear energy safer by transitioning from nuclear fission towards nuclear fusion. Fossil energy continues to exist but continuously reduces its share of total energy and electricity production.

Transport is a mixture of public and private transport. The state invests in developing and updating the public means of transport, especially trains, electric buses, undergrounds, and tramways. There are public subsidies for the e-mobility industry. Electric vehicles operated as private and public means of transport are continuously becoming more important and cheaper. In urban areas, private and public transport companies offer ride-sharing through shared e-bikes, e-scooters, and e-cars. They offer pay-per-ride as well as subscription services at a flat rate.

***Scenario 4: Participatory digital ecology – Participatory digital democracy and society’s ecology.*** In this scenario, climate change is seen as a major societal problem, and there are lots of efforts for trying to bring about a carbon-free economy that is socially equitable and benefits all citizens. Renewable energy has become by far the dominant form of energy. 90 per cent of all produced energy and electricity stems from renewable energy sources. Sustainable energy production is considered as being a major public concern.

In this scenario, a new understanding of growth and degrowth has emerged. Growth is not growth of corporate profits but focuses on the growth of wealth for all and productivity that benefits all under sustainable conditions. A successful green transition has been made possible through the democratisation of climate and energy policy. Climate assemblies, which are citizens’ assemblies that take place regularly to discuss the issue and gather information and ideas on what a green future might look like, have been established. In such assemblies, groups such as citizens, self-managed companies, environmental and other civil society groups, governments, unions, etc. discuss and plan how a sustainable future can best be achieved. Climate change and environmental degradation are global problems. Therefore, there are global climate assemblies and environmental forums that bring together relevant actors at the international level in order to discuss and formulate binding international agreements on sustainability issues. They have worked on the foundations of a global constitution that involves environmental, democratic, social, and other issues. The United Nations act as global institution that organises these assemblies, debates, and policies. The goal of this international collaboration is to promote global welfare within planetary boundaries, whereby growth is managed at a global societal level so that it primarily occurs where there are unmet needs. Questions of how social, economic, and environmental development affect different parts of the world are part of these international debates and agreements. The goal is to overcome uneven global and geographical development.

Public and co-operative energy companies dominate the environmental economy, focusing on the generation of wind power, solar energy, and hydroelectricity. At the communal, regional, and national levels, public energy companies exist. The public owns and operates them with the defined public remit of advancing the production of green energy that is widely available and highly affordable.

Green energy co-operatives are operated at the municipal level. All households living in the municipality collectively own them. The defined goal of energy co-operatives is to fully base energy production on renewable sources and make it available to and affordable for everyone. The co-operative is a self-managed democratic organisation where the households that are members of the co-operative and the workers elect the management that is accountable to the community of members and workers. The municipality provides the basic investment for such an energy co-operative by investing a certain amount of money per household. Thereby, each household becomes a member of the co-operative. The invested money is used for creating solar panels and wind turbines on local land that the municipality owns. The community partly consumes the produced green energy locally and sells the rest on the energy market. The energy co-operatives operate as not-for-profit organisations. When surplus revenues are generated, they are paid out as a co-operative dividend to the members (households and workers), not in the form of money but energy cost discounts. The households that are members of the co-operative commit to buy their energy from the co-operative.

Energy co-operatives cooperate with each other (co-operation of energy co-operatives) and with the public energy sector (public/co-op energy partnerships) with the goal of making green energy widely available to everyone at affordable prices.

Given that citizens possess widespread environmental awareness and consciousness, society is capable of containing the escalation of environmental problems. Climate change is significantly slowed down by coming closer to a carbon-free economy. Nature is better preserved and less polluted. Human-made natural disasters have become much less frequent.

There is a move away from the inbuilt obsolescence of digital devices. Many software and hardware companies are now not-for-profit co-operatives that reject greenwashing and engage in the advancement of true social and environmental sustainability. As a consequence, green computing devices have become common, which have reusable parts and updatable hardware components. This new digital model has significantly reduced the amount of annual e-waste to an average of 1 kg per capita. Facilities recycle almost all e-waste and authorities document it.

There is a move from the capitalist big data economy towards the co-operative small data economy. Data management is highly decentralised. Platforms and devices are privacy-friendly by design, which means that they use data minimisation by default. They do not store massive amounts of private data for corporate and state purposes, but only store the data that is necessary for operating devices. Lots of data is stored locally on users’ devices and is, based on transparent and meaningful informed consent, shared with selected other users. The amount of data and meta-data generated is reduced by half. The size of data centres decreases significantly. As a consequence, operating green digital devices, platforms, and networks consumes less energy than before. In addition, the vast share of the required energy stems from renewable sources.

In our scenario, the state has launched a public transport offensive. Public and co-operative not-for-profit companies operate public transport, which makes transport cheap and affordable. Where possible, public transport is freely available. There is a general requirement to make public transport as affordable as possible. Public transport is one of the pioneers in the use of green means of transport. It predominantly utilises electric vehicles (trains, buses, tramways) powered by renewable energy sources. Citizens continue to use private cars, but their share in the total transport flows has declined, and they are all based on renewable energy sources.

The urban/rural transport divide has been significantly reduced by large public investments into the public transport infrastructure, where not just existing infrastructure was modernised but also a large amount of new public transport infrastructures was created in rural areas. Thereby, public transport has also become widespread based on dense rural public transport networks with frequent connections.

E-bikes, e-scooters, and e-cars are widely offered as common means of transport that can be rented for free or at a small fee. Such services are operated by both public transport companies and transport co-operatives that are not-for-profit organisations. Users of such e-vehicles register with their personal data, and authorities monitor usage to avoid thieves stealing or vandals deliberately damaging the publicly shared vehicles.

### STEEP-P: Digital political futures

***Scenario 1: Authoritarian digital politics – Representative digital authoritarianism’s politics.*** In this scenario, the political system is a classical one-party dictatorship that has outlawed all opposition and has executed or imprisoned all critics and opponents. The political system uses violence and terror as general political principles. There is a charismatic leader who rules society as a dictator. There are no elections. The political system is a one-party system led by one person. Three big tech corporations control the economy. The CEOs of these three corporations are personally and politically closely aligned with the dictator and key political functionaries in the authoritarian system. The dictator is a rich former CEO of a large oil company. He sees the ideal society as a CEO monarchy where a CEO rules the state in an absolute manner and organises many of its aspects like a corporation. While private corporations largely operate educational, welfare and social services, the state maintains a very active repressive role in organising inner and outer defence, especially surveillance, control of citizens, censorship, policing, prisons, the military, and secret services. Policies that protect human and civil rights have been uprooted.

There are no news media organisations that report critically on the government. They were all dissolved after the ruling party took power. The regime either imprisoned or executed critical journalists and editors. The corporation that is owned and run by the CEO, who is also the minister of economic, financial, social, and cultural affairs, holds monopolies in the economic fields of e-commerce, entertainment, culture, finance, manufacturing, and health. It owns all television and radio stations, cinemas, newspapers, and the major entertainment and news-oriented Internet platforms. A private news and entertainment monopoly exists. The system places a strong focus on tabloid news and entertainment. News programmes take on a tabloid character. They scandalise, simplify, spread fake news and misinformation, use lots of colour images and very little text, and continuously construct scapegoats that are presented as enemies of society. They are propaganda machines. Attempts to publish factual news and critical opinions are violently repressed by the police and secret services.

CCTV cameras and facial recognition technologies monitor all public life on a large scale. Surveillance drones patrol the skies and monitor citizens from above. Mobile devices and smart technologies have become embedded in the human body so that every citizen can constantly be tracked and monitored. A constant citizen scoring system is in place, it uses algorithms based on large-scale surveillance to monitor and score all citizens. Those who actively support and promote the ruling party and ideology in all aspects of everyday life are rewarded with high scores. The algorithms lower the score for behaviour such as idleness, conversations critical of the dominant ideology and party, accessing prohibited information that is critical of the dictatorship, attempts to organise meetings, events, or campaigns that could result in criticism of the system, etc.

Neural interfaces monitor the thoughts of all citizens. Therefore, negative citizen scores are also assigned for critical ideas and thoughts that are not aligned with the dominant ideology. The citizen score ranges on a scale between 0 and 100. Those who have scores above 95 are rewarded with free holidays at a seaside resort, monetary payments, and free luxury goods such as expensive cars. 50 is a critical threshold. Those whose score drops below 50 are classified as absolute enemies of the state and society who deserve capital punishment. The rule of law does not exist. The system conducts no trials, not even show trials. When a citizen’s score drops below 50, there is an automatic execution system that results in the killing of the citizen and his or her family. The execution methods are unknown in advance and are constantly enhanced and made ever more horrible. They include, for example, robotic and drone-based firing squads, detonating brain chips, the release of toxic chemicals from implants, public hangings, and live-streamed beheadings. A particularly feared execution method is the nightmare method.

The neural interfaces that are implanted into all human brains are capable of scanning dreams, whereby they scan citizens’ nightmares and identify their worst fears. In the nightmare method of execution, a personalised method of killing is developed that makes the affected individual’s worst nightmare and fear become reality.

***Scenario 2: Participatory authoritarian digital politics – Participatory digital authoritarianism’s politics.*** In this scenario, digital technologies are widely used, presented by the government as democratic participatory innovations that, however, in reality, stepwise make society more illiberal, undemocratic, and dictatorial. The government incentives/encourages citizens to become active members/collaborators in spreading pro-government discourse. This can involve state-funded creators or influencers promoting favourable messages or engaging with users to promote the government. Political influencers who are critical of the government exist and are not censored. However, government positions are much more visible in the public sphere than critical voices. Pro-government influencers are well-paid by government programmes, while critical political influencers tend to be precarious digital workers who lack the resources necessary for gaining large attention in the digital public sphere.

Digital plebiscites play an important role in the political system. The ruling party presents the political system as highly democratic and participatory. The government decides on what topics plebiscites are held and how its questions are formulated. Citizens decide using an e-voting app. The questions are often formulated in a biased manner. Plebiscites are accompanied by government propaganda campaigns in favour of certain options so that there is no fair representation of different opinions in the public sphere. An example is the introduction of the death penalty and the deportation of all immigrants who do not hold the national citizenship.

The government held two digital plebiscites on these topics, justifying them with the claims that violent crime had exploded dramatically and immigrants were responsible for major social problems. Before the two votes, the president hosted a popular daily online talk show with phone-in possibilities. He presented manufactured cases of violent crimes allegedly committed by immigrants and asked citizens to phone in and voice their opinions. The talk show team preselected only xenophobic callers supportive of the government position, who were then put live on air. There was always one pseudo-critic who argued against the president, utilising poor arguments that were easily dismantled by the president. The pseudo-critic was in fact always a functionary of the ruling party disguised as an ordinary citizen. Thereby, the regime created the impression of an open debate that in fact was highly manipulated. Government opinion was furthermore supported by fake opinion poll results that were invented but presented to the public as credible research.

Participatory digital democracy functions as tokenistic and the regime limits it to trivial matters at the national level. For example, the government used a crowdsourcing app asking citizens to submit poster designs for public campaigns praising the president and his party. This crowdsourced political public relations campaign made sure that critical poster designs could be submitted but were never selected. The campaign was merely an exercise in political propaganda disguising itself as participatory democracy.

News media that publish critical voices on the government in their opinion sections exist and are not censored. However, there is a range of state-controlled media and private government-friendly media that receive a large amount of press funding. Critical media do not receive such public funding, which is why they can only employ a few journalists and have problems investing in outreach activities. Consequently, there is a wide range of news media in the public sphere, but the latter is strongly dominated by pro-government reporting. Government-friendly media are dominated by light entertainment programmes. News is almost exclusively tabloid news organised as infotainment with a strong pro-government bias.

***Scenario 3: Representative digital democracy – Representative digital democracy and society’s politics.*** This scenario is based on a representative democracy that uses digital technologies for engaging citizens in the form of open governance. The government has invested lots of public money into the development of e-voting technologies. As a result, a new e-voting app was developed and introduced at the national and local levels. The app combines blockchain technology and cryptography in order to make e-voting compatible with the demands for privacy, security, transparency, and scalability. The e-voting system respects four key design principles of e-voting: voter privacy (secrecy of the ballot), election security (the cast votes are not altered, the voting system is accessible and operational, votes are genuine and verified), procedural transparency (the voting process is transparent to the public and the voters, the results can be audited by experts and laypersons), and scalability (the voting system can handle a large number of votes and can operate over a longer time period).

The government created an open data portal where all government data and documents are published and maintained. There are tools that visualise the data and provide summaries in textual and visual formats to citizens. The government release a transparency report on all of its activities, budget spending, and contracts. The government remains highly transparent and accountable. There is a public performance dashboard where citizens can, in an easy manner, display to what extent the government has achieved defined objectives.

Mechanisms of open governance have strengthened public debate and citizen-to-government communication. They result in debates and recommendations, not in citizens’ direct formulation or voting on policies. This means that the political system is a representative democracy engaging citizens via new technologies and not a direct democracy where citizen decisions replace elected representatives.

The government regularly organises town hall meetings, policy dialogues, and citizen juries online and offline in order to obtain citizens’ inputs on policy questions, organise exchanges between government officials and citizens, and gather feedback. Policy dialogues take place online and offline. They are informal debates between the government, citizens, civil society, businesses, and other stakeholders. Citizen juries invite randomly selected citizens and groups to review and discuss policy questions and formulate policy recommendations. They take place online and offline. Town hall meetings are online, offline, and hybrid meetings where the government directly connects to and discusses policy issues with citizens.

US and Chinese digital giants that operate major Internet platforms (Alphabet’s Google and YouTube; Meta’s Instagram, Facebook, and WhatsApp; ByteDance’s TikTok; Elon Musk’s X; the video streaming platforms Netflix, Amazon Prime and Disney+) dominate the digital media landscape. Alternatives include several not-for-profit Internet platforms and platform co-operatives that, in comparison to the digital giants, have a small number of active users. Public service media organisations are focused on broadcasting. They have media players such as the BBC iPlayer and Internet presences on US and Chinese social media platforms, but do not operate their own Internet platforms. The main measure taken against fake news, disinformation, and misinformation spread on social media platforms that dominate the digital media world is regulation. Especially, data protection laws for safeguarding privacy are used in order to force corporate Internet platforms to respect users’ privacy. The overall situation of the digital media world is that there are a handful of Internet platforms that control the vast share of Internet visits, which are regulated by national and regional data protection laws that are partly ignored by the platforms.

QTube (Q standing for Questions) is the parliament’s own YouTube-like platform that supports Prime Minister’s Questions (PMQs). PMQs is a session of the House of Commons held every Wednesday at noon where the British Prime Minister answers questions posed by members of parliament. The way PMQs has been communicated to the public via the media changed over the years since it was introduced on July 18, 1961 ( The Independent, 2011): The earliest media practice for covering the event was that journalists sat in the audience and took notes by hand. In 1978, a BBC radio broadcast of PMQs was introduced, and in 1989, televised coverage began. Today, British citizens can also watch PMQs over the Internet on the BBC iPlayer and as live or archived broadcasts on various websites, which gives them more spatio-temporal flexibility and makes PMQs not just a national, but a global political event.

In our scenario, the government implemented the QTube platform that is based on an idea first introduced by Christian Fuchs (2014) as part of the UK House of Commons Speaker’s Commission on Digital Democracy. In its report on digital democracy, the Speaker’s Commission on Digital Democracy characterised QTube as an idea designed to “help to increase the accountability of Ministers to the public”; it recommended that “The House of Commons should experiment with new ways of enabling the public to put forward questions for ministers” ( Speaker’s Commission on Digital Democracy, 2015, 46).

The British Parliament did not introduce QTube. In our scenario, the idea was taken up and introduced by parliament in order to enhance citizen-to-parliament communication and make representative democracy more deliberative via digital communication.

In our scenario, the parliament adopted QTube together with Prime Minister’s Questions. It works the following way:

• Parliament operates its own video-sharing platform that is specifically designed for PMQs.
• From Wednesday after PMQs ends until Sunday evening, citizens can upload user-generated short videos to the platform. Each video asks one question to the Prime Minister. The videos can be generated and uploaded by single citizens or groups of citizens who engage in producing them, e.g. as part of community centres, school classes, university modules, political debate clubs, etc.
• Letting users create short videos for PMQs is not just a concrete practice in citizen-centred politics and political citizen engagement, but an incentive for citizens and groups of citizens to be creative: Some of the videos are quite basic, whereas others are artistic, and again others use excerpts from videos that show current political events that are reused and remixed in various ways. Images, news bites, sounds, art, etc, are likely used in creative and unpredictable ways.
• There is a fixed maximum duration of each video. Videos that are longer than the maximum time cannot be uploaded and are automatically rejected by the electronic system.
• On Monday and Tuesday, the system closes the upload possibility and goes into the voting mode. All uploaded videos are presented in a user-friendly way on QTube, and citizens can vote on them. Voting could either work in such a way that each IP address in the UK has one vote and is technically excluded from further votes, or that citizens are enabled to vote by registering with their personal data and can log into the system, which allows them to cast one vote each.
• Content that violates human rights or propagates authoritarianism is not put to a vote.
• The videos that achieve the highest number of votes win the selection process. There is a fixed number of user-generated videos per week of around 3–5, which is selected this way.
• The citizens whose videos are selected are invited to be present at PMQs and to sit in one of the front benches.
• The user-generated questions are broadcast on a large screen in the parliamentary chamber. The videos are intermixed with the regular questions asked by MPs.

QTube’s overall effect is that parliamentary debates are enhanced by citizen voices. Representative democracy has become more based on deliberative digital democracy.

The representative digital democracy in our scenario is both substantive and procedural. It employs innovative forms of open governance procedures, such as QTube, to promote citizen engagement in governance and democratic discussion. It is substantive because it values digital democratic constitutional rights highly. At the national level, constitutional rights have been updated to suit the digital age by including a digital democracy section in the constitutional framework, based on and enshrining the principles of the *European Declaration on Digital Rights and Principles for the Digital Decade* ( European Union 2023). The key principles of the Declaration have been incorporated as constitutional amendments: digitality should be people-centric, solidaristic, inclusive, fair, participatory, safe, secure, empowering, and sustainable; and should promote digital connectivity, digital education, fair and equitable digital working conditions, digital public services, human-centric, trustworthy, and ethical AI, as well as data privacy and protection. Our scenario of representative digital democracy and society embodies the ethos of the European Declaration on Digital Rights and Principles for the Digital Decade:

“The EU way for the digital transformation of our societies and economy encompasses in particular digital sovereignty in an open manner, respect for fundamental rights, rule of law and democracy, inclusion, accessibility, equality, sustainability, resilience, security, improving quality of life, the availability of services and respect of everyone’s rights and aspirations. […] putting people at the centre of the digital transformation; supporting solidarity and inclusion, through connectivity, digital education, training and skills, fair and just working conditions as well as access to digital public services online; restating the importance of freedom of choice in interactions with algorithms and artificial intelligence systems and in a fair digital environment; fostering participation in the digital public space; increasing safety, security and empowerment in the digital environment, in particular for children and young people, while ensuring privacy and individual control over data; promoting sustainability” ( European Union 2023).

One issue our digital democratic society faces is that strong constitutional digital rights are enshrined in the digital constitution, yet dominant US and Chinese Internet platforms often ignore or actively undermine them. There is a contradiction between democratic ideals defined by law at the national level and the economic reality dominated by the digital giants.

***Scenario 4: Participatory digital democracy – Participatory digital democracy and society’s politics.*** In this scenario, there is a participatory democracy where citizens play a key role in political decision-making. The model does not limit democracy to the political system but extends it to society and its various aspects, such as the workplace, education, and culture. There are general elections at the municipal, national, and regional levels where citizens elect councils and parliaments. This representative system of democracy is one part of the political system that interacts with a second part that is strongly based on a combination of participatory and deliberative democracy.

Participatory, deliberative and constitutional democracy are combined, which means that public political debates and citizen engagement are encouraged and supported under the premise that they have to respect human and civil rights guaranteed by a democratic constitution. All local councils have introduced participatory services, including participatory budgeting. Free and open-source platforms such as Decidim ( https://decidim.org/) and Consul ( https://consuldemocracy.org/) are widely used as tools for municipal digital participatory budgeting. Participatory budgeting fuses online and offline debates and participation. There is a strong focus on developing citizens’ (e-)participation skills, practising participation, and learning participation by doing it. Routinely, a share of twenty per cent of the local budget is used for (e-)participatory budgeting. (e-)participation is meaningful, which means proposals are suggested and discussed that make a difference to citizens’ lives and are not tokenistic.

In our scenario, participatory budgeting is prioritised as an important measure of participatory democracy. “Participatory budgeting allows ordinary people to identify priorities, propose projects, and vote on how government funds are spent, typically at the municipal level. This can be achieved through the use of various tools. For example, digital tools such as apps can be used to connect citizens, thereby facilitating collective problem identification. The goal is to increase civic engagement, transparency, and government accountability while ensuring that public spending reflects the needs of the community” ( Fuchs et al. 2025, 118). This means that the system avoids the so called park-bench problem, where the state limits citizen engagement to trivial matters such as what colour park benches should have.

The government has introduced structural reforms that are intended to support participatory and deliberative democracy. The state reduced the working week from 40 to 32 hours with full wage compensation, which has freed up time for citizens outside of labour time. Four additional annual public holidays, so-called Democracy Days, were introduced. On these days, there are special democratic events all over the country, such as citizen assemblies, town hall meetings, and neighbourhood forums where hot political topics are debated. The public budgets for education at all levels were significantly increased, a measure that was combined with educational reforms so that political and democratic education was introduced as a subject in all secondary schools. There is a wide variety of adult education possibilities, including the opportunity for adults who have been active in working life for some time to combine work with higher education. The general approach is that the government tries to combine reforms that bring about good living conditions with offers and opportunities for political debate, citizen engagement, and educational opportunities.

There is a vivid civil society with a wide range of civil society and non-government organisations dedicated to social and democratic issues. A network of tech activists and civil society groups, including Wikipedia and the free and open-source movement, has developed and operates civic tech technologies that enhance deliberative and participatory digital democracy. A variety of democracy-enhancing tools are available. The Digital Democracy App is a hub that links to and combines such digital democracy tools. Wikipedia organises the Digital Democracy App as a free software tool and a network of developers and citizens continuously improves it. It is independent of capital, government, and ideology. Its features include Democracy Forums, a popular citizen-to-citizen communication tool where citizens debate on a variety of issues at the local and translocal level. There are also online petition tools and organisational tools supporting social movements and citizens’ campaigning. The Digital Democracy App has together with structural reforms helped to bring about a vivid democratic public sphere.

The FactCheckApp was introduced and is widely used by citizens. A network of independent fact-checking organisations operates the app. The FactCheckApp automatically imports all parliamentary speeches and debates, as well as public statements of politicians and public opinion influencers (for example, lobbyists, corporate public relations, social media influencers, etc.). The app utilises a combination of AI-based fact-checking algorithms and a network of highly educated independent fact-checkers and journalists, who have secure employment conditions, for checking the correctness of such public statements. The app publishes reports on public statements together with fact-checking reports. There is a wide public interest in and the adoption of the app. When factually incorrect information is discovered, those making the claims are invited to respond. The response, respectively, the information that there is no response, is published next to the fact-checking report.

Society’s dynamic public sphere has also brought about a structural transformation of the media landscape. Public service media and not-for-profit citizen media and media co-operatives have become more important. For-profit entertainment and news media continue to exist, but they are of less interest, and there is less presence of tabloid media that is sensationalist, privilege entertainment over news and education, simplifies, and is politically biased. There is entertainment in the media, but it is now less important, so that professionally produced news, documentaries, educational, and art programmes are more important. Quality media outweighs tabloid media. Public service media are well funded by a licence fee paid by households and companies, independent from governments, corporations, and ideology, and digital innovators creating new digital information services that appeal to all citizens, including the younger generation. The transformed media landscape and public sphere have managed to significantly reduce the amount of fake news, echo chambers, disinformation, misinformation, deep fakes, algorithmic politics, digital authoritarianism, online hatred, AI-generated propaganda, etc. The emergence of a variety of public service Internet platforms and platform co-operatives has resulted in a significant reduction of the interest in and the influence and power of the former big digital tech and media monopolists, such as Alphabet/Google, Meta/Facebook, TikTok/ByteDance, Amazon, Apple, Netflix, Disney, etc.

Public service media have introduced Club 2.0, a popular deliberative digital democracy tool that is a digital version of a format introduced by the Austrian Broadcasting Corporation (ORF), Austria’s public service broadcaster, in 1976. It was broadcast regularly until 1995. In the UK, a similar format was called After Dark and was broadcast on Channel 4 and the BBC.

Club 2 was an open-ended, uncensored live debate programme that became known for its fearless approach to controversial issues and outspoken guests. Each episode unfolded as a live studio discussion with no predetermined time limit, allowing conversations to develop naturally and without interruption. The programme’s format was deliberately simple yet distinctive: there was no studio audience, only a single moderator and a small group of four to eight diverse guests gathered in a relaxed, living room setting. This intimate atmosphere encouraged honest, spontaneous, and often heated exchanges that made Club 2 one of the most provocative and engaging programmes on television. Club 2 was a public sphere and deliberative democracy in action, supported by public service media. The rise of reality TV put more emphasis on entertainment and drove back such formats.

**Figure 2.**
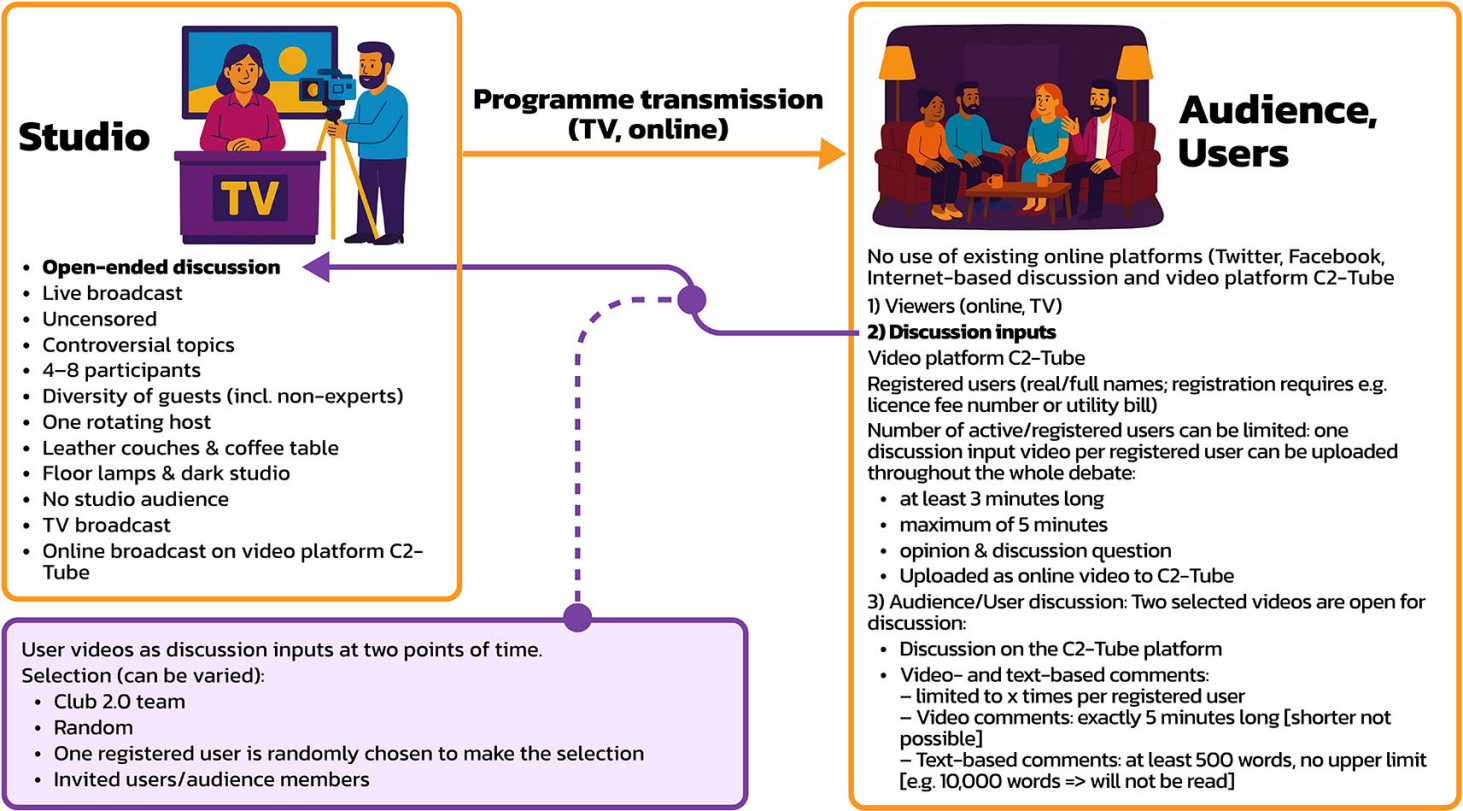
The concept of the Club 2.0 platform, a public service Internet platform for deliberative and participatory digital democracy.

Media reform, combined with a strong public support of public service media, brought about the introduction of Club 2.0, the digital renewal of Club 2. Figure 2 visualises how Club 2.0 works.

• “Club 2.0 uses and extends the traditional principles of Club 2. The television broadcast is based on the tried and tested Club 2 rules, which are crucial to the quality of the format. Club 2.0 broadcasts are open-ended, live, and uncensored. […] Club 2.0 is a cross-medium that combines live television and the Internet, […] Club 2.0 is broadcast live online via a video platform. […] Club 2.0 […] [uses] its own online video platform […]: C2-Tube allows viewers to watch the debate online and via a range of technical devices. […] It is possible for users to generate discussion inputs and for these to be actively included in the programme. This characteristic is linked to a non-anonymous registration of users on the platform. […] User-generated discussion inputs should preferably have a video format. […] At certain times during the live broadcast, a user-generated video is selected and shown as input for the studio discussion. In such videos, users formulate their opinions on the topic and can also introduce a discussion question. In a two- to three-hour discussion, about two to three such user-generated inputs could be used. It is inevitable that a selection mechanism will be used to decide which user-generated videos will be shown in the live broadcast. There are several ways to do this, such as random selection, selection by the production team, selection by a registered user determined at random, selection by a special guest, etc. […] Club 2.0 allows users to discuss the programme topic with each other. The discussion can take place during and/or after the live broadcast. The selected videos that function as discussion inputs can be released for discussion on C2-Tube. […] Club 2.0 is integrated into educational institutions where people learn and create knowledge together by elaborating discussion inputs and collective positions and producing them in video form. This requires that the topics of certain Club 2.0 programmes are known somewhat in advance. This can be achieved by publishing a programme of topics. Groups of users can prepare videos together, which they can upload to the platform on the evening of the relevant Club 2.0 programme as soon as the upload option is activated. [..] Club 2.0 is a Club 2 in the age of the Internet and digital platforms. Club 2.0 is a manifestation of the democratic digital public sphere. It combines elements of deliberative and participatory democracy“( Fuchs, 2024, 379–382).

Club 2.0 relies on fact-checking software to provide context to the presented opinions of the guests and the submitted videos of the audience members.

A series of democratic innovations has enhanced the public sphere and deliberative democracy. For example, there is the regular use of citizen assemblies, where randomly selected citizens deliberate and make recommendations on public issues. They meet both online and offline.

Deliberative polling is used in politics. It is a method of public opinion research that combines random sampling with in-depth deliberation to understand what people would think about an issue if they were better informed and had time to discuss it with others. There is a random sample of citizens who are surveyed on a particular topic. They receive balanced briefing materials outlining different sides of the issues. The citizens are invited to meet for one day and discuss the topic in a structured manner. The participants continue the discussion online for another week. Experts also provide input and participate in these hybrid discussions. After the discussion, participants are surveyed again to see how their opinions have changed. The results are published and widely debated in the media, public events, and parliament.

There is a constant development of and experimentation with new forms of digital democracy, such as online citizen assemblies, digital town halls, digital citizen juries, deliberative polling, local councils and neighbourhood platforms, e-participation platforms, etc. Government and civil society together have created a culture where there is a strong focus and interest in digital democratic innovations. An important guiding principle is that digital democracy is organised and practised as online/offline hybrids so that citizens can build trust face-to-face and democracy becomes less anonymous. Digital participation is supported by face-to-face meetings and events.

Just like in scenario 3, our scenario 4 also emphasises constitutional digital democracy by redeveloping the democratic constitution based on the principles of the *European Declaration on Digital Rights and Principles for the Digital Decade* ( European Union 2023) so that the democratic constitution features a section on digital democratic rights that emphasises strong protections of human and basic rights in the digital age so that it is “putting people at the centre of the digital transformation” ( European Union 2023).

There are constitutional provisions for digital equality, universal and affordable digital access, advanced digital skills development in education, digital workers’ rights, digital public services, human-centred and ethical AI, users’ rights, online consumer protection, data protection and privacy, digital cultural diversity, and a sustainable information society, among others. Given that in the past, the dominant US and Chinese big tech companies ignored or undermined digital democracy and digital rights, policymakers in our scenario are not content with merely defining digital rights. They have expanded the principle of participatory democracy to the digital economy itself, resulting in new democratic organisational models of digital companies and platforms. These models challenge the power of big tech and have become widely used and successful. Notably, these organisational models include non-commercial, not-for-profit platform co-operatives, public service Internet platforms, free, libre, and open source software (FLOSS), the creative and digital commons movement, and federated, decentralised platforms that, together, form the Fediverse, alongside digital public/commons partnerships. Such organisations not only adopt a different economic approach compared to entities like Google, YouTube, Instagram, TikTok, Amazon, Apple, Alibaba, etc., but also are actively committed and legally obliged to uphold the principles of the digital democratic constitution.

## Concluding remarks on the future of digital society

This paper explored the futures of digital democracy and society by 2050 using a realist science fiction approach. The future is unpredictable, but we created four distinct speculative scenarios that serve as imaginative tools for critical reflection. We presented four scenarios of digitalisation and society, focusing particularly on the impact of these developments on digital democracy. Methodologically, we combined the scenario technique with STEEP analysis to conceptualise the four scenarios and five societal dimensions of each one. It is worth making a few remarks about the probabilities and drivers that could push us towards one or another of these scenarios.

As noted in the introduction, the non-deterministic and complex nature of social systems means that we can only attempt to approximate, not predict, what societies will look like in 5, 10, 20 or more years. As society’s development is not mechanically determined, but rather shaped by relative chance, we cannot calculate the probability of future developments or make probabilistic statements associated with each scenario. As Immanuel Wallerstein points out, “the world of 2050 will be what we make it”, thereby leaving “full rein for our agency, for our commitment, and for our moral judgement” ( Wallerstein, 1998, 64). However, he also acknowledges that “which choice the participants collectively will make” about society’s future “is inherently unpredictable” ( Wallerstein, 2004, 77). In this context, Marx stressed that society’s development is shaped by the interaction of societal conditions and social struggles that together constitute the logic of relative chance: “Men make their own history, but they do not make it just as they please; they do not make it under circumstances chosen by themselves, but under circumstances directly encountered, given and transmitted from the past” (Marx, 1852, 103).

We want to make some remarks on how to prevent the authoritarian developments illustrated in scenarios 1 and 2. Resisting or counteracting the drift towards autocracy requires a strategy that works on different pillars: Three important strategies are a) anti-fascist and anti-authoritarian social movements; b) structural reforms of (digital) democracy and society. Together, these measures can make democracy more resilient; and c) constitutional mechanisms.

Let us have a look at an example from Germany in 2024. The nonprofit newsroom Correctiv reported that the AfD organised a meeting where mass deportations of immigrants were discussed under the euphemistic term of “remigration”. In the wake of these revelations, more than three million people across various German cities ( Sander and Ansa, 2024) took to the streets to demonstrate against the AfD. As a notable result of these demonstrations, the “AfD dropped below 20% for the first time since July [2023], with voters citing nationwide demonstrations against the far-right as the most important issue” ( Rinke and Steitz, 2024), thereby illustrating that public protests can exert a far-reaching political influence.

Achieving a resilient democracy necessitates a new approach: “Resilient democracy is a process where democrats produce and reproduce structures of democratic resilience that condition democratic action that again reproduce and produce structures of democratic resilience.” ( Fuchs 2025, 8).

Furthermore, the term of resilient democracy can be thought of as something that cannot simply be achieved but rather defined as something dynamic, “we can understand and define democracy’s resilience as actors in society, such as citizens, governments, political parties, social movements, or non-governmental organisations, creating mechanisms that resist the transformation of society from a democracy into a dictatorship or actively and successfully resisting such a transformation. Resilient democracy has a subjective and objective dimension; it is both actor-and systems-based and involves both the subjective features of practices and ideas as well as objective structures. Resilient democracy requires humans committed to democracy who, as democratic actors, create democratic structures and mechanisms that resist dictatorships and dictatorial tendencies and try to institutionally shield democracy against its breakdown and transition into dictatorship. Actors in resilient democracy involve institutions, parties, citizens, and civil society/social movements.” ( Fuchs 2025, 9).

As political theorist Benjamin Barber (2006) has emphasised, although digital media has the potential to further democratic participation, it also has negative impacts in this regard that need to be taken into account when theorising the future of digital democracy and society. In particular, Barber warns against 1) the rapid pace of digital discourse, 2) the unmediated dissemination of digital information, 3) the fragmented and private structure of Internet communities, 4) the digital divide, and 5) the corporations’ monopolisation of digital platforms. Barber notes that these five “configurative elements of the Internet […] go against what is required of a democratic system” ( Barber 2006, 1). Such dangers remain relevant in a future participatory digital society but will be easier to deal with than today.

However, another danger is that participatory digital democracy can turn into participatory digital authoritarianism and participatory digital fascism. In this context, Barber (1997) warned: “Plebiscitary democrats will be mindlessly enthralled by interactive instant polling and imagine a time when private consumers make precedent-shattering public choices with no more serious thought than they give to which button to hit when they are surfing a hundred-channel cable system” ( Barber 1997, 223). There is the “danger of the potential usage of televoting for installing push-button and point-and-click decision systems that give legitimacy to authoritarian leadership that manipulates public opinion“( Fuchs 2008, 236). A recent example of this danger can be seen in Elon Musk’s use of Twitter/X polls following his acquisition of the platform in 2022. He conducted a series of high-profile polls (on reinstating banned accounts, including Donald Trump’s, and on his continued leadership of the platform), framing the results as the authentic voice of the people and invoking the Latin phrase “vox populi, vox dei” ( https://x.com/elonmusk/status/1594131768298315777). However, these polls were restricted to Twitter/X’s self-selected user base, and Musk retained complete control over which questions were asked, when they were asked, and whether he would act on the results. What appeared to be direct democratic participation was, in practice, a legitimising spectacle in service of the decisions of a single, powerful individual.

Therefore, understanding (digital) democracy merely as deliberative and participatory (digital) democracy is not enough. Democracy needs to ensure that the basic rights of citizens are not violated so that, for example, referenda, mini-publics, citizens’ initiatives, social movements, participatory budgeting, e-participation, the digital public sphere, etc. do not extend to undemocratic practices that violate basic and human rights. Therefore, digital democracy, deliberative and participatory digital democracy, e-participation, and the digital public sphere need to be combined with constitutional democracy that defines and protects democratic constitutional rights in the context of the Internet. Building on Habermas, we can say that digital democracy requires the combination of “communicative forms of democratic opinion- and will-formation” and institutionalised “constitutional principles” ( Habermas 1996, 298).

We now want to summarise the four presented scenarios:

### Scenario 1: Representative digital authoritarianism

This scenario depicts a repressive dictatorship where culture is dominated by the ruling ideology – education, media, and art serve as propaganda tools and critical thought is banned. The regime instrumentalises digital technology for surveillance and control: advanced dataveillance, implanted sensors and brain interfaces constantly monitor citizens. The economy operates as a state-monopolised and capitalist system: a few party-aligned tech conglomerates dominate all industries, while the state denies worker rights and alternative ownership. Environment policy is neglected – fossil fuels and nuclear power are embraced, renewable energy is outlawed, and e-waste is rampant under endless consumerism. Politically, a single-party headed by a strongman rules with absolute power; elections and dissents are eliminated and terrorised, and digital authoritarianism turns technology into a means to control the populace.

### Scenario 2: Participatory digital authoritarianism

Here, a pseudo-democratic culture allows limited debate but stigmatises dissent – state propaganda and social pressure (“woke virus”) suppress critical voices even as citizens feel superficially free to speak. The regime uses digital platforms for controlled engagement: citizens vote in tightly managed, propagandised plebiscites and online polls. The economy is largely capitalist with minimal regulation: oligopolies and public/private-partnerships enrich regime-aligned corporations, while social scoring and gamified apps nudge citizens into regime-friendly behaviours. Ecologically, the state funds nuclear and fossil infrastructure (often as crony deals) and only tokenistically backs “green” projects for profit, leading to continued climate harm. In politics, a veneer of democracy hides authoritarianism: digital plebiscites and e-voting exist but are rigged to legitimise the ruling party, exemplifying how plebiscitary democracy can serve a charismatic autocrat.

### Scenario 3: Representative digital democracy

In this scenario, a pragmatic culture values education for both jobs and citizenship – learners use a mix of traditional and digital tools in schools, and a diverse media landscape exists, though private platforms drive it. Technology is regulated to strengthen governance: e-government, e-voting and open-data platforms make public services transparent and responsive, protected by strong privacy and data laws. The economy is a mixed welfare capitalism: unions and co-operatives exist alongside private firms, with progressive taxation and public services that aim to reduce inequality. Ecology is a public priority – the state invests in renewables (public wind and solar companies) and regulates industry to cut emissions, moving steadily toward carbon reduction. Politically, representative democracy prevails: elected bodies govern, while citizens contribute through digital consultations, town halls, and transparency portals. Digital democracy tools enhance accountability (e.g. online participatory budgeting or platforms like QTube), yet power remains with elected representatives.

### Scenario 4: Participatory digital democracy and society

This scenario imagines a highly active civic culture: schools and communities emphasise critical thinking and co-determination, and a vibrant civil society and media commons flourish. Technology is human-centred and open: privacy-enhancing infrastructures, civic-tech apps, and commons-based digital platforms empower genuine citizen engagement (e.g. online deliberation and fact-checking tools). The economy is democratised: a majority of enterprises are public, co-operative or nonprofit, worker self-management is the norm, automation is used to eliminate menial labour (with guaranteed retraining), and wealth is widely shared. Ecologically, society is committed to sustainability – almost all energy is renewable and managed by public/co-op utilities, e-waste is minimised through reuse, and digital networks run on green power. In politics, participatory and deliberative democracy reigns: elected governments exist, but many decisions emerge from informed citizens’ assemblies, participatory budgeting and constant online/offline dialogue. This lively digital democracy underscores how collective self-government and technology can co-evolve, making governance transparent, inclusive and constitutionally grounded.

**Figure 3.**
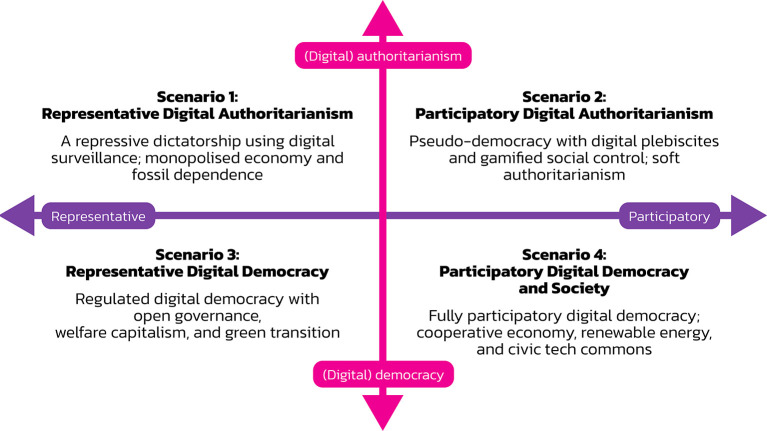
Four future scenarios for digital society.

These four scenarios are based on the STEEP-driven scenario framework for digital democracy (the user’s text) and informed by research on digital authoritarianism and digital democracy, which highlight how technology can either empower citizens or entrench autocracy. Table 3 and Figure 3 summarise key features of the four scenarios.

**Table 3.** Four future scenarios for digital society.

Dimension	Scenario 1: Representative Digital Authoritarianism	Scenario 2: Participatory Digital Authoritarianism	Scenario 3: Representative Digital Democracy	Scenario 4: Participatory Digital Democracy & Society
Culture	Propaganda, indoctrination, censorship, ideology of obedience	Pseudo-pluralism, social pressure, “woke” scapegoating	Mixed culture: free but commercialised public sphere	Critical, inclusive, creative civic culture and media commons
Technology	Surveillance tech, brain interfaces, dataveillance	Public/private partnerships for controlled engagement	E-government, e-voting, open-data transparency	Commons-based civic tech, privacy-enhancing tools, FLOSS ecosystems
Economy	State monopolies aligned with the regime; no worker rights	Crony capitalism; oligopolies; gamified citizen control	Regulated welfare capitalism with unions & co-ops	Democratic mixed economy: co-ops, commons, fair automation
Ecology	Fossil fuel dominance; outlawed renewables; extreme pollution	Fossil/nuclear subsidies; tokenistic greenwashing	Public–private green energy transition; renewables growing	Fully renewable, public/co-op energy system; green digital infrastructure
Politics	Digital authoritarianism, no elections, terror & censorship; one-party dictatorship; total surveillance; leader rule	Managed plebiscitary autocracy with digital legitimation; controlled participation via manipulated plebiscites	Accountable representative democracy with open governance; representative democracy with digital transparency	Deep participatory & deliberative democracy with empowered citizens; participatory & deliberative democracy combining online/offline participation

## Ethics and consent

Ethical approval and consent were not required.

## Data and software availability

No data was collected for this article, which is why no accompanying data is available.
