# Supplementary material for: The Futures of Digital Democracy: Four Scenarios
Source: Open Res Eur. 2026 Jun 4;6:49. Originally published 2026 Feb 13. [Version 2] doi: 10.12688/openreseurope.22910.2 (PMC13087570; doi:10.12688/openreseurope.22910.2)
Supplement: Peer review history

# Reviewer report: Reviewer response for version 2

**DOI:** 10.21956/openreseurope.26068.r75455

**Published:** 2026-06-26

**Authors:** Orsolya Johanna Zsigmond-Sziebig PhD LLM

**Affiliations:** 1 University of Szeged, Szeged, Hungary

Based on the authors' answers and the paper's unique vision, I believe the article is suitable for indexing.

 In summary, I accept the modifications to the article, and the authors successfully included a more specific introduction while retaining the original framework of the paper and still incorporating most of the proposed considerations.

Is the argument persuasive and supported by appropriate evidence?

Partly

Is the work clearly and cogently presented?

Yes

Is the topic of the essay discussed accurately in the context of the current literature?

Partly

Does the essay contribute to the cultural, historical, social understanding of the field?

Yes

Reviewer Expertise:

NA

I confirm that I have read this submission and believe that I have an appropriate level of expertise to confirm that it is of an acceptable scientific standard.

# Reviewer report: Reviewer response for version 2

**DOI:** 10.21956/openreseurope.26068.r75457

**Published:** 2026-06-05

**Authors:** Stephan Lewandowsky

**Affiliations:** 1 University of Bristol, Bristol, UK

I am happy with the revisions.

Is the argument persuasive and supported by appropriate evidence?

Partly

Is the work clearly and cogently presented?

Partly

Is the topic of the essay discussed accurately in the context of the current literature?

Yes

Does the essay contribute to the cultural, historical, social understanding of the field?

Yes

Reviewer Expertise:

Cognitive science; democracy and cognition; online technology and cognition; misinformation

I confirm that I have read this submission and believe that I have an appropriate level of expertise to confirm that it is of an acceptable scientific standard.

# Reviewer report: Reviewer response for version 1

**DOI:** 10.21956/openreseurope.24770.r69838

**Published:** 2026-04-16

**Authors:** Davide Grossi

**Affiliations:** 1 University of Groningen, University of Amsterdam, Amsterdam, The Netherlands

-------

 It is undoubtedly a very timely exercise to try and map plausible scenarios concerning the near-future evolution of the interface of democracy and digital technologies. The added value of such an exercise lies in raising awareness over the possible long-term effects of processes that are already at play in the present of our societies. The paper accomplishes this goal nicely and it is certainly a worthwhile contribution. It especially succeeds, in my view, in outlining what our ideal scenario should be for us to strive for as a society, when it comes to digital democracy.

 Having said the above, the paper does have in my view a number of blind spots and would benefit from improved clarity on a number of points, first and foremost its methodology. Below I list a number of concrete suggestions, which I hope can be useful to the authors for producing the next iteration of the manuscript.

  

1. Broad methodological points.

The paper puts a strong emphasis, already in the abstract, on the use of specific methods (Scenario Development Technique and STEEP Analysis) in developing its contribution. However, these methods obtain little coverage in the paper (only a few relatively vague words are dedicated to them on pages 6 and 7). I recommend this issue to be addressed before publication as it is important for the reader to understand how the authors proceeded with the study and arrived at their conclusions: this is the most important aspect that underpins the choice of a methodology. A few more concrete points, and suggestions, concerning methodology follow:

 - p. 3: There is an immediate focus on four scenarios. The fact that they are four is somewhat motivated by the focus on two dimensions (depicted in Figure 1). However, the authors do not elaborate on why they decided to focus on these, rather than other dimensions, or at least this is not clearly articulated in the text.

 - p. 6: The ‘scenario technique’ is introduced, essentially by providing a definition of what is understood as a scenario in the paper. This makes sense, but what I missed there is the actual methodological aspect: by which process are scenarios created? The paper mentions the “critical uncertainties method”, but this method is not explained. As a consequence, the reader is left wondering about what approach has been used in the paper and how the authors arrived at the four scenarios that ended up being presented. It would be important to clarify this aspect ahead of publication.

 - p. 6: The ‘STEEP’ method is presented and introduced in some more details, which is nice. But, again, it would be important for the reader to know how the authors arrived at Table 2, elaborating the earlier work by Fisher et al, which is referred to in the text. In general, the point that would be important to clarify are the arguments that led to the features identified in the table rather than others and, therefore, the extent to which Table 2 could be critically contested.

 - The paper differentiates the description of the four scenarios, from their analysis via STEEP. However, the ways in which scenarios are introduced and described is already phrased in the context of STEEP. So, in a way, STEEP is used to *describe* the scenarios, rather than *analyse* them. I think this is fine, but I found it confusing at first given that the methods are explicitly introduced as different and separated. I invite the authors to clarify this point.

  

1. Broader comments to improve clarity and presentation.

- One small suggestion about the title. The title fits the content but could also be understood as overstating the content of the paper. Perhaps the addition of “four scenarios” would make for a better description of the content of the paper. So, something like: “The Futures of Digital Democracy: Four Scenarios”.

 - Throughout the paper: long quotes are rendered in different ways, sometimes with emphasis and centred text (e.g., p.6) and sometimes in running text with normal indentation (opening quote in Introduction, or on p.7). It may be advisable to make the presentation of long quotes homogeneous.

 - Throughout the paper: both ‘sociocultural’ and ‘socio-cultural’ occur in the paper. Please harmonise.

 - p. 3: The second paragraph lays a connection between non-determinism, complexity and free will. It should be noted that unpredictability could be observed already in deterministic complex systems, without the need for free will. In general, I am not convinced the quote from Wallerstein actually helps positioning the paper. It actually confuses the line of argument I find.

 - p. 3: There is a reference to Aristotle & Lord, 2013. Referred in this way, it appears as if these are two authors. Please correct.

 - p. 9, first paragraph. Two periods in the second-last sentence: “..”

 - p. 9: Description of the first scenario. “Most of content consists of the regime leader’s speeches, writings and ideas”. Perhaps a plural “leaders’ ” may make better sense here. The scenario mentions ‘alt-right’ learning platforms. In principle, the description is a description of a techno-totalitarian society. As such, it could fit a right- as well as a left-extremist ideology, so I am unsure whether this emphasis on ‘right’ is granted. Perhaps the authors’ argument should be expanded on this point.

 - p. 10. In the 4th scenario, the authors emphasise how humanities and social-sciences education is integrated into STEM programmes so that “the STEM subjects develop a greater awareness of societal problems”. I cannot agree more with this ideal vision of the future of education. At the same time, it points to a blind-spot in my view. It is absolutely true that in a participatory digital culture, STEM education would need to grow the awareness the authors mention. At the same time, a process in the opposite direction needs to take place too: students of the humanities and social sciences would need to develop deeper awareness of scientific and technological trends, and feel actually at home, to a good extent, in STEM areas. What can be achieved in terms of participatory ideas has to be rooted on the awareness of what is technologically possible. In a participatory digital democracy, students of the social sciences and humanities would be exposed to a targeted STEM education too, towards mathematical and computational literacy. I invite the authors to consider this two-way traffic in their thinking.

 - p. 10. The 4th scenario sometimes mentions very detailed technological solutions (unlike in the other scenarios). For example: “The digital patient record includes a function …” I wonder what the rationale for such a design actually is, and if pros and cons of it have been considered (perhaps are these discussed in relevant literature?). In general, I wonder whether this level of detail on highly speculative interventions actually serves the purpose of the scenario description. A similar level of detail appears on p. 15 discussing the “Automation Impact Assessment” legislation (120 days, 45 days). A reader might wonder how the authors arrived at those numbers. The same comment applies to the data mentioned on page 17. In general, it is totally fine to come up with speculative data for the purpose of the exercise the authors engage with, however, it would be interesting for a reader to understand how those numbers are arrived at.

 - p. 11. When discussing Scenario 1 there is an emphasis on the fact that privacy legislation has been uprooted. However, in that kind of scenario even more fundamental legislation must have been uprooted such as conflict-of-interest legislation or anti-trust legislation. In general, the scenario outlines a society in which all the checks-and-balances infrastructure of modern democracies has been uprooted. Perhaps this needs to be mentioned as privacy legislation is not the only legislation that has to go to make Scenario 1 possible.

 - p. 12: totalitarianism and fascism are mentioned next to one another. It is unclear whether the authors intend them as synonyms or as different phenomena that might run in parallel in the society of Scenario 1. Please clarify.

 - p. 15: a number of major economic players of the 2010s and 2020s are mentioned explicitly (first column). However, this is an exercise focusing on 2050 so it is debatable whether using those companies in the scenario makes sense. Some of them are likely to perhaps not exist anymore. Perhaps some words of qualification about this point might be useful. Of course the authors’ argument to use those companies as analogies is clear, but perhaps the analogy should be mentioned explicitly. I was unfamiliar with Guru and there seem to be several companies with that name. The same comment applies at page 20, last paragraph, or page 22 second column.

 - p. 16: “four days and 32 hours”. Is this meant to mean 32 hours work per week in at most 4 days?

 - p. 16: GenAI is mentioned prominently here. However, by 2050 it will be a technology of the past (already nowadays there appears to be a pivot towards what the hype calls ‘agentic AI’ … just to mention an example). I wonder whether it wouldn’t serve the paper better to phrase the description in terms of future AI technologies that were enabled by the GenAI advent in the 2020s.

 - p. 16: “The reason is that robots and AI systems do not have emotions, feelings, etc.” This is stated as a fact by the authors, but it is a highly debated matter both within AI and within philosophy. This is arguably the case now (I say arguably because even that might be debated), but whether AI couldn’t implement those functions in principle is much more doubtful. A well-established position on the philosophy of computation and AI is that humans are also computers (on a biological substratum) and they have also been programmed (by evolution). A quote often attributed to Von Neumann (from a 1948 talk): “You insist that there is something that a machine can't do. If you will tell me precisely what it is that a machine cannot do, then I can always make a machine which will do just that.” Much has been written on the topic, so this type of statements are too sweeping. Perhaps the authors might find this volume from the 80s (so, much preceding the current AI hype) interesting: Graubard, S. (1988). The artificial intelligence debate. *False, Starts, Real Foundations, Massachusetts: The MIT Press, Cambridge*.

 - p. 16. Notice also that the sentence “they act merely based on software programmes, algorithms and databases” has some redundancy: software programmes are algorithms implemented on digital computers.

 - p. 19: The notion of “digital plebiscite” is used. I found it a suggestive and to-the-point notion. However, it would be good to perhaps explain more explicitly what the authors understand with it. Are they a sort of instant digitally-mediated referenda with yes/no answers?

 - p. 21: Add links to the sites of Decidim and Consul. Perhaps also add a reference to what is understood under participatory budgeting (e.g., Smith’s book on Democratic Innovations, or more recent work of the authors’ choice).

 - p. 23: Please add a relevant reference when discussing Deliberative Polling.

Is the argument persuasive and supported by appropriate evidence?

Partly

Is the work clearly and cogently presented?

Partly

Is the topic of the essay discussed accurately in the context of the current literature?

Partly

Does the essay contribute to the cultural, historical, social understanding of the field?

Yes

Reviewer Expertise:

NA

I confirm that I have read this submission and believe that I have an appropriate level of expertise to confirm that it is of an acceptable scientific standard.

# Response

**Published:** 2026-05-19

**Authors:** Christian Fuchs

**Response to Review 3**

 We found all three first round reviews very helpful. They have helped us to create an improved second version.

**Review 3: Comment from Reviewer:**

 The paper puts a strong emphasis, already in the abstract, on the use of specific methods (Scenario Development Technique and STEEP Analysis) in developing its contribution. However, these methods obtain little coverage in the paper (only a few relatively vague words are dedicated to them on pages 6 and 7). I recommend this issue to be addressed before publication as it is important for the reader to understand how the authors proceeded with the study and arrived at their conclusions: this is the most important aspect that underpins the choice of a methodology. [...] - p. 6: The ‘scenario technique’ is introduced, essentially by providing a definition of what is understood as a scenario in the paper. This makes sense, but what I missed there is the actual methodological aspect: by which process are scenarios created? The paper mentions the “critical uncertainties method”, but this method is not explained. As a consequence, the reader is left wondering about what approach has been used in the paper and how the authors arrived at the four scenarios that ended up being presented. It would be important to clarify this aspect ahead of publication. - p. 6: The ‘STEEP’ method is presented and introduced in some more details, which is nice. But, again, it would be important for the reader to know how the authors arrived at Table 2, elaborating the earlier work by Fisher et al, which is referred to in the text. In general, the point that would be important to clarify are the arguments that led to the features identified in the table rather than others and, therefore, the extent to which Table 2 could be critically contested.

**Answer by the authors:**

 We added a paragraph that discusses how table 2 came about:

 “STEEP stands for the initials of these dimensions. Table 2 gives an overview of these five aspects. In this paper, STEEP is used for analysing each of our four scenarios in more detail. This is because each scenario is a type of society and all societies have all of the STEEP dimensions as their subsystems. Table 2 shows example aspects of what belongs to each of the four dimensions. The examples are based on Fisher *et al.* (2020). We added further example dimensions.”

 In respect to the critical uncertainties method, we added the following paragraph:

 “We used the critical uncertainties method for creating a Cartesian system with two axes that combine the mode of societal organisation as one axis and the form of governance as the other axis.”

**Review 3: Comment from Reviewer:**

 - p. 3: There is an immediate focus on four scenarios. The fact that they are four is somewhat motivated by the focus on two dimensions (depicted in Figure 1). However, the authors do not elaborate on why they decided to focus on these, rather than other dimensions, or at least this is not clearly articulated in the text.

**Answer by the authors:**

 We have explained why these two axes are so important:

 “Our model’s two dimensions, the distinction between democracy/authoritarianism and between participation/representation, reflect fundamental questions of political theory, namely governance and society’s mode of organisation.”

**Review 3: Comment from Reviewer:**

 "One small suggestion about the title. The title fits the content but could also be understood as overstating the content of the paper. Perhaps the addition of “four scenarios” would make for a better description of the content of the paper. So, something like: “The Futures of Digital Democracy: Four Scenarios”.

**Answer by the authors:**

 We changed the title to “The Futures of Digital Democracy: Four Scenarios”.

**Review 3: Comment from Reviewer:**

 - p. 3: The second paragraph lays a connection between non-determinism, complexity and free will. It should be noted that unpredictability could be observed already in deterministic complex systems, without the need for free will. In general, I am not convinced the quote from Wallerstein actually helps positioning the paper. It actually confuses the line of argument I find. **Answer by the authors:**

 We added the argument that unpredictability is not just a feature of society but can also be found in nature: “Wolfgang Hofkirchner (2011) says that the world is neither a determine clockwork nor an unpredictable cloud. He stresses that nature has “the freedom to choose between several alternatives which make up a non-devoid space of possibilities, compared with mechanical systems where there is only one possibility” (Hofkirchner 2011, 190)”.

**Review 3: Comment from Reviewer:**

 When discussing Scenario 1 there is an emphasis on the fact that privacy legislation has been uprooted. However, in that kind of scenario even more fundamental legislation must have been uprooted such as conflict-of-interest legislation or anti-trust legislation. In general, the scenario outlines a society in which all the checks-and-balances infrastructure of modern democracies has been uprooted. Perhaps this needs to be mentioned as privacy legislation is not the only legislation that has to go to make Scenario 1 possible.

**Answer by the authors:**

 We have added a general remark that rights have been undermined to scenario 1:

 “Policies that protect human and civil rights have been uprooted.”

**Review 3: Comment from Reviewer:**

 “four days and 32 hours”. Is this meant to mean 32 hours work per week in at most 4 days?

**Answer by the authors:**

 We changed the formulation to “the working week has been reduced from 40 to 32 hours”

**Review 3: Comment from Reviewer:**

 The notion of “digital plebiscite” is used. I found it a suggestive and to-the-point notion. However, it would be good to perhaps explain more explicitly what the authors understand with it. Are they a sort of instant digitally-mediated referenda with yes/no answers?

**Answer by the authors:**

 We added a definition:

 “A digital plebiscite is an instant digitally-mediated referendum with yes/no answers”

# Reviewer report: Reviewer response for version 1

**DOI:** 10.21956/openreseurope.24770.r70899

**Published:** 2026-04-09

**Authors:** Orsolya Johanna Zsigmond-Sziebig PhD LLM

**Affiliations:** 1 University of Szeged, Szeged, Hungary

The essay entitled “ *The futures of digital democracy*” is an interesting and sometimes thought-provoking paper that examines different scenarios regarding digitalisation and society. The theme is important, as in the last couple of years, we have seen the rise of artificial intelligence (AI) and digital solutions. As a lawyer, I have a very strict, sometimes meticulous approach to research, so this time an exception was made to provide a review that considers the authors’ background and the paper’s style. I believe the submitted essay meets academic standards, but I recommend minor changes for consideration.

 The research questions are understandable, but a stronger introduction would help to identify the exact scope of the research. For me, it was not clear why Williams’ work on the future was chosen as the frame for the article. The authors accepted it without any criticism, so it would be useful to highlight the importance of the framework.

 The original aim of the authors was to provide a “science fiction” story about the future of digital democracy. In my opinion, this topic can be discussed in academic writing less hypothetically, as there is a growing body of literature on digitalisation, AI, democratic values, and society. To support and clarify the scenarios, the authors should cite academic sources and contemporary examples to strengthen their arguments. Today, it is clear how governments, political actors, and organisations are harnessing the potential of digital solutions and social media platforms. In this context, it would also be possible to link specific concepts with real-world examples.

 Among the four established scenarios, Participatory Digital Democracy and Society are portrayed in an overly utopian manner, and it is clear from the authors’ perspective that they view it as the indisputably most advantageous option. But the literature shows that digitalisation has drawbacks even under the most favourable circumstances. An example of this is when digital solutions are aimed at promoting participatory democracy.

 The digital ecological futures section of the STEEP-E analysis feels a bit overlooked and doesn't go beyond generalising existing information. I think the starting point of this chapter is already on the wrong track, as it has now been proven that, within a couple of decades, fossil fuels are expected to run out this century, so it is hard to imagine that even authoritarian regimes will use them since we will have to face the depletion of gas and oil reserves within a few decades. Moreover, training and producing artificial intelligence consumes a huge amount of drinking water, so hopefully, green-minded states will realise the disadvantages of digital technologies, rather than relying solely on them. It is virtually impossible to predict what technological innovations we can expect in the coming decades, given that new alternatives in green technologies emerge almost every year. Among other things, the choice between alternatives such as electric vehicles and alternative fuels based on CCUS (Carbon Capture, Utilisation, and Storage) technologies will determine the future of the green solutions implemented and developed. Given the significant differences in government and political systems among the international community, it would have been worthwhile to address this issue and consider whether the protection of green and environmental concerns is feasible only under democratic conditions. If this is the authors’ stance, then why do they believe that authoritarian regimes are hesitant to support green issues?

 Overall, I found the essay interesting; the research is new, and I have never read anything similar. But, based on the above, I would formulate some recommendations for consideration.

 1. Strengthening the introductory chapter to provide a more precise understanding of the research and the goals of the authors with special regard to the accepted and applied reserach frameworks and methodology.

 2. In the concrete analysis of different scenarios, I would apply a more realistic and a less science fiction approach, as the topic can rely on valid and real-life examples and literature.

 3. Rethink and rewrite the “ecology” chapter of the STEEP analyses.

Is the argument persuasive and supported by appropriate evidence?

Partly

Is the work clearly and cogently presented?

Yes

Is the topic of the essay discussed accurately in the context of the current literature?

Partly

Does the essay contribute to the cultural, historical, social understanding of the field?

Yes

Reviewer Expertise:

International and European Environmental Law with special regard to the following: wildlife crime, ecocide, human rights, green, sustainable human settlements, SDGs

I confirm that I have read this submission and believe that I have an appropriate level of expertise to confirm that it is of an acceptable scientific standard, however I have significant reservations, as outlined above.

# Response

**Published:** 2026-05-19

**Authors:** Christian Fuchs

***Response to Review 2***

*We found all three first round reviews very helpful. They have helped us to create an improved second version.*

*******Reviewer 2: Comment from Reviewer:***

*For me, it was not clear why Williams’ work on the future was chosen as the frame for the article. The authors accepted it without any criticism, so it would be useful to highlight the importance of the framework.*

****

***Reviewer 2: Comment from Reviewer:***

*The original aim of the authors was to provide a “science fiction” story about the future of digital democracy. In my opinion, this topic can be discussed in academic writing less hypothetically, as there is a growing body of literature on digitalisation, AI, democratic values, and society. To support and clarify the scenarios, the authors should cite academic sources and contemporary examples to strengthen their arguments. Today, it is clear how governments, political actors, and organisations are harnessing the potential of digital solutions and social media platforms. In this context, it would also be possible to link specific concepts with real-world examples. …. In the concrete analysis of different scenarios, I would apply a more realistic and a less science fiction approach, as the topic can rely on valid and real-life examples and literature.*

***Answer by the authors:***

*Our paper is not a regular academic paper but more a piece of realist science fiction, which requires a writing style that differs from traditional academic papers. However, we have taken the reviewer’s advice into account by adding references to literature about participatory budgeting and the real-world example of digital human rights by referencing the European Declaration on Digital Rights and Principles for the Digital Decade.*

***Reviewer 2: Comment from Reviewer:***

*Among the four established scenarios, Participatory Digital Democracy and Society are portrayed in an overly utopian manner, and it is clear from the authors’ perspective that they view it as the indisputably most advantageous option. But the literature shows that digitalisation has drawbacks even under the most favourable circumstances. An example of this is when digital solutions are aimed at promoting participatory democracy.*

***Answer by the authors:***

*We added a discussion of the dangers and limits of participatory democracy to the conclusion. We argued that there is the danger that participation turns into anti-democratic mechanisms, which is why constitutional safeguards against authoritarianism and fascism are needed.*

*******Reviewer 2: Comment from Reviewer:***

*The digital ecological futures section of the STEEP-E analysis feels a bit overlooked and doesn't go beyond generalising existing information. I think the starting point of this chapter is already on the wrong track, as it has now been proven that, within a couple of decades, fossil fuels are expected to run out this century, so it is hard to imagine that even authoritarian regimes will use them since we will have to face the depletion of gas and oil reserves within a few decades. Moreover, training and producing artificial intelligence consumes a huge amount of drinking water, so hopefully, green-minded states will realise the disadvantages of digital technologies, rather than relying solely on them. It is virtually impossible to predict what technological innovations we can expect in the coming decades, given that new alternatives in green technologies emerge almost every year. Among other things, the choice between alternatives such as electric vehicles and alternative fuels based on CCUS (Carbon Capture, Utilisation, and Storage) technologies will determine the future of the green solutions implemented and developed. Given the significant differences in government and political systems among the international community, it would have been worthwhile to address this issue and consider whether the protection of green and environmental concerns is feasible only under democratic conditions. If this is the authors’ stance, then why do they believe that authoritarian regimes are hesitant to support green issues?*

***Answer by the authors:***

*We had already outlined aspects of a green authoritarianism/fascism under the ecological dimension of the participatory digital authoritarianism-scenario. Furthermore, we now added a description of how the representative digital authoritarianism-scenario deals with ecological questions:*

*“The privileged elite and party members are living in zones less affected by climate change. Regime critics and scapegoated minorities are left in or deported to high-risk areas where there is flooding, natural disasters, etc. where many of them die. This is an ecological form of getting rid of the regime’s opponents.* *The assertions by political opponents that fossil fuels have a definitive expiration date are dismissed by the ruling regime as fake news. Renewable energy sources continue to be delegitimised as purely ideologically driven, while fossil fuels are promoted and subsidised under the guise of national tradition. Although the regime is internally aware that current resources are finite, it maintains a lack of transparency, categorising any reports to the contrary as misinformation. Simultaneously, a gradual strategic pivot toward expanded nuclear energy is underway.* *By strategically centring the national discourse on nuclear energy, the regime further delegitimises renewable alternatives. This shift serves a dual purpose: it functions as both a rhetorical justification and empirical proof for the regime’s platform, effectively providing the necessary leverage to silence or marginalise political dissidents”.* *We also extended the discussion of the environmental aspects of the participatory digital authoritarianism scenario, arguing that the regime conducts a plebiscite on energy futures.*

*Furthermore, we extended the discussion of environmental aspects in the section on participatory digital democracy, arguing that climate assemblies play an important role in the scenario.*

**Reviewer 2:**

 Strengthening the introductory chapter to provide a more precise understanding of the research and the goals of the authors with special regard to the accepted and applied research frameworks and methodology.

**Answer by the authors:**

 We provided an introductory paragraph that defines the paper’s purpose.

# Reviewer report: Reviewer response for version 1

**DOI:** 10.21956/openreseurope.24770.r70609

**Published:** 2026-03-28

**Authors:** Stephan Lewandowsky

**Affiliations:** 1 University of Bristol, Bristol, UK

The authors present 4 future scenarios for digital democracy or autocracy (2 of each) and explore the implications of each scenario using the STEEP framework. That is, they explore (1) sociocultural factors, (2) technological factors, (3) economic factors, (4) ecological factors, and (5) political and legal factors. The scenarios are plausible extremes along two dimensions (from autocracy to democracy, and from participatory to representative). 

 I enjoyed reading the article because it was quite creative in its presentation of the scenarios, and the exploration within STEEP was informative. There are however at least two important omissions that a revision should address:

 (1) At present, nothing is said about the drivers that may push us into one or the other of those scenarios. What is the path from today's world to those 4 scenarios, and how might one counteract the drift towards autocracy? How can we facilitate participatory democracy? Some exploration of our agency in this process would be helpful.

 (2) Relatedly, what are the probabilities associated with each scenario? Can we make such probabilistic statements?

 In addition to addressing those omissions, a revision should also consider the following points:

 1. The introduction meanders in places and would benefit from a sharper focus. A clearer statement of purpose and scope early on would help orient the reader.

 2. The analysis and description of digital fascism is interesting and novel (to me at least).

 3. There is some redundancy that could be addressed through tighter editing. On page 5, for instance, the eight types of future scenarios are explained at a level of detail that is not fully warranted and takes up too much space.

 4. The authors should clarify how the Williams taxonomy relates to what they actually did: as it stands, the taxonomy is introduced but its relationship to the authors' own analytical choices is left underspecified.

 5.  Finally, Figure 3 largely duplicates the information already conveyed in Figure 1. I suggest dropping 1 and replacing it with 3. 

Is the argument persuasive and supported by appropriate evidence?

Partly

Is the work clearly and cogently presented?

Partly

Is the topic of the essay discussed accurately in the context of the current literature?

Yes

Does the essay contribute to the cultural, historical, social understanding of the field?

Yes

Reviewer Expertise:

Cognitive science; democracy and cognition; online technology and cognition; misinformation

I confirm that I have read this submission and believe that I have an appropriate level of expertise to confirm that it is of an acceptable scientific standard, however I have significant reservations, as outlined above.

# Response

**Published:** 2026-05-19

**Authors:** Christian Fuchs

**Response to Review 1**

 We found all three first round reviews very helpful. They have helped us to create an improved second version.

**Reviewer 1: **At present, nothing is said about the drivers that may push us into one or the other of those scenarios. What is the path from today's world to those 4 scenarios, and how might one counteract the drift towards autocracy? How can we facilitate participatory democracy? Some exploration of our agency in this process would be helpful.

**Answer by the author: **We added an argumentation to the conclusion that addresses this remark. The conclusion now argues for focusing on social struggles for democracy, discusses the notion of resilient democracy, and stresses the need for democratic constitutionalism.

**Reviewer 1: **Relatedly, what are the probabilities associated with each scenario? Can we make such probabilistic statements?

**Answer by the author:** We supplemented the conclusion with the argument that, as societal development is determined by relative chance, we cannot calculate the probability of future developments. What we can address, however, is how to prevent the authoritarian developments illustrated in scenarios 1 and 2. This relates to the points made in the previous remark.   

**Reviewer 1: **The introduction meanders in places and would benefit from a sharper focus. A clearer statement of purpose and scope early on would help orient the reader.

**Reviewer 1:** The authors should clarify how the Williams taxonomy relates to what they actually did: as it stands, the taxonomy is introduced but its relationship to the authors' own analytical choices is left underspecified.

**Answer by the authors: **We added the following introductory sentence: “Our approach to conceptualising scenarios for the future development of digital democracy and society is informed by the cultural theorist Raymond Williams's analysis of writing about the future.”

 However, with regard to how Williams’ taxonomy relates to our analysis, the original draft already contained a paragraph explaining this connection in detail. This paragraph outlined how we based our four scenarios on Williams’ criteria of “willed transformation” and “technological transformation”, as well as their utopian and dystopian dimensions. “Williams has a particular interest in human-centred and technological utopias and dystopias. For us, the combination of both holds special interest. The technological dimension is interesting for us because we intend to write about digital futures. Human-centredness is important because we are interested in writing about the future of human society on Earth. Therefore, there are no extraterrestrial beings such as Martians in our scenarios, and we focus on life on Earth and not on other planets. Human-centred stories also allow us to focus on potential futures of contemporary power structures. In our scenarios, power structures continue to exist but have been altered by humans wilfully as the outcome of certain social struggles. Therefore, our scenarios are manifestations of the lower part of Table 1 that focuses on human-centred utopias and dystopias as well as technological utopias and dystopias. Our digital futures scenarios ask what happens when we combine the technological and the human-centred storytelling with the question of how the futures of digital society could look.”

**Reviewer 1: **Finally, Figure 3 largely duplicates the information already conveyed in Figure 1. I suggest dropping 1 and replacing it with 3. 

**Response by the authors:** We do not see it as feasible to drop figure 1 or figure 3 as they provide good visual summaries of the paper.
